# Supplementary figures and images for: Experiences of women with Zika virus (ZIKV) versus the provision of health services in two cities in Colombia: A qualitative study
Source: PLoS One. 2021 Dec 2;16(12):e0260583. doi: 10.1371/journal.pone.0260583 (PMC8638867; doi:10.1371/journal.pone.0260583)

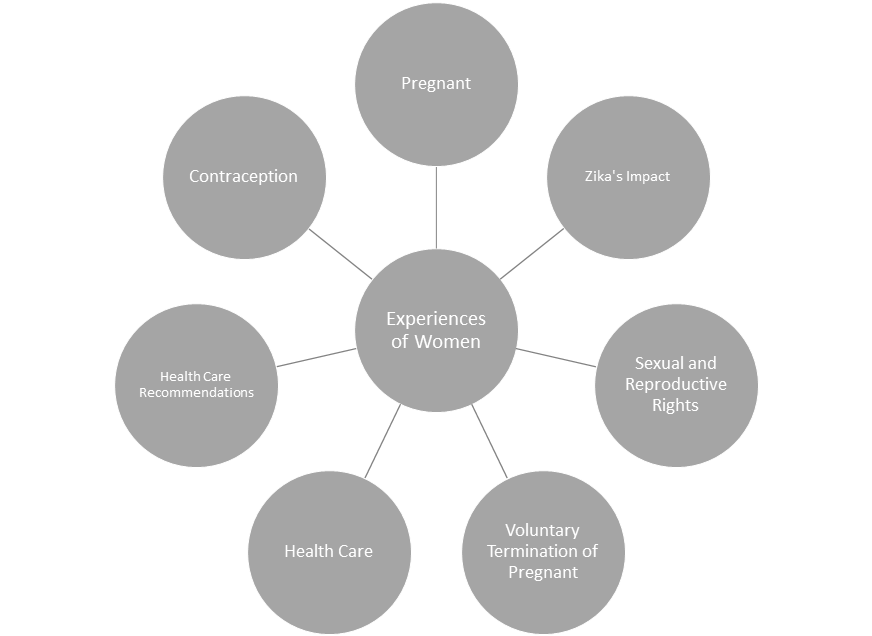

Supplement: S1 Fig — (TIF) [file pone.0260583.s001.tif]
