# Supplementary material for: Experiences of women with Zika virus (ZIKV) versus the provision of health services in two cities in Colombia: A qualitative study
Source: PLoS One. 2021 Dec 2;16(12):e0260583. doi: 10.1371/journal.pone.0260583 (PMC8638867; doi:10.1371/journal.pone.0260583)
Supplement: S1 Table — (DOCX) [file pone.0260583.s002.docx]

| **Interviewee No.** | **Age during pregnancy** | **Level of Education** | **Marital Status** | **Occupation** | **Socio-economic Level** | **Trimester of pregnancy at diagnosis of ZIKV** | **Birth Outcomes** |
| --- | --- | --- | --- | --- | --- | --- | --- |
| Interviewee 1 | 24 | Complete Basic Secondary Education | Single | Self-employed | Middle-Middle | First Trimester (Month:1) | Microcephalia |
| Interviewee 2 | 23 | Complete Basic Secondary Education | Married | Higher education student | Middle-Middle | First Trimester (Month:3) | Voluntary Interruption of Pregnant |
| Interviewee 3 | 24 | Complete Basic Secondary Education | Single | Unemployed | Low-High | Third Trimester (Month:8) | Congenital Malformations |
| Interviewee 4 | 31 | Higher Education - University | Married | Chef | High | First Trimester (Month:2) | Voluntary Interruption of Pregnant |
| Interviewee 5 | 23 | Higher Education - Technical | Single | Occasional work | Middle-High | First Trimester (Month:3) | Healthy Children |
| Interviewee 6 | 32 | Complete Basic Secondary Education | Common Law Marriage | Self-employed | Middle-Middle | Second Trimester (Month:5) | Perinatal Death |
| Interviewee 7 | 34 | Higher Education - University | Married | Household work | High | Third Trimester (Month:8) | Intra-uterine death |
| Interviewee 8 | 34 | Higher Education - University | Married | Financial work | High | Second Trimester (Month:4) | Perinatal Death |
| Interviewee 9 | 23 | Higher Education - University | Married | Health care work | High | “Before Pregnant” (not clear about the month of diagnosis) | Intra-uterine death |
| Interviewee 10 | 33 | Higher Education - University | Single | Higher education student | Middle-Middle | Second Trimester (Month:5) | Premature Healthy Children |
