# Supplementary material for: Experiences of women with Zika virus (ZIKV) versus the provision of health services in two cities in Colombia: A qualitative study
Source: PLoS One. 2021 Dec 2;16(12):e0260583. doi: 10.1371/journal.pone.0260583 (PMC8638867; doi:10.1371/journal.pone.0260583)
Supplement: S2 Table — (DOCX) [file pone.0260583.s003.docx]

| **Interviewee No.** | **Age during pregnancy** | **Level of Education** | **Marital Status** | **Occupation** | **Socio-economic Level** | **Trimester of pregnancy at diagnosis of ZIKV** | **Birth Outcomes** |
| --- | --- | --- | --- | --- | --- | --- | --- |
| Interviewee 11 | 22 | Complete Basic Secondary Education | Married | Household work | Middle-Low | First Trimester (Month:3) | Microcephalia |
| Interviewee 12 | 35 | Higher Education - Technical | Married | Self-employed | Middle-Middle | Third Trimester (Month:8) | Healthy Children |
| Interviewee 13 | 27 | Incomplete Basic Secondary Education | Single | Household work | Low-High | Without symptoms | Voluntary Interruption of Pregnant |
| Interviewee 14 | 37 | Complete Basic Secondary Education | Married | No Data | Low-High | First Trimester (Month:1) | Blighted Ovum |
| Interviewee 15 | 21 | Complete Basic Secondary Education | Married | Waitress | Middle-High | Second Trimester (Month:4) | Perinatal Death |
| Interviewee 16 | 28 | Higher Education - University | Married | Household work | Middle-Middle | Without symptoms | Microcephalia |
| Interviewee 17 | 31 | Higher Education - Technical | Married | Nursing | Middle-Middle | First Trimester (Month:3) | Healthy Children |
| Interviewee 18 | 20 | Complete Basic Secondary Education | Single | Household work | Middle-High | Second Trimester (Month:6) | Hydrocephalus |
| Interviewee 19 | 19 | Higher Education - Technical | Married | Household work | Middle-High | First Trimester (Month:2) | Healthy Children |
| Interviewee 20 | 34 | Complete Basic Secondary Education | Married | Self-employed | Middle-Middle | First Trimester (Month:3) | Dandy–Walker malformation |
| Interviewee 21 | 21 | Higher Education - University | Single | Higher education student | Low-High | First Trimester (Month:3) | Healthy Children |
| Interviewee 22 | 32 | Complete Basic Secondary Education | Divorced | Self-employed | Middle-High | First Trimester (Month:1) | Perinatal Death |
