# Supplementary material for: Experiences of women with Zika virus (ZIKV) versus the provision of health services in two cities in Colombia: A qualitative study
Source: PLoS One. 2021 Dec 2;16(12):e0260583. doi: 10.1371/journal.pone.0260583 (PMC8638867; doi:10.1371/journal.pone.0260583)
Supplement: S1 File — (DOCX) [file pone.0260583.s004.docx]

| Universidad de los Andes |
| --- |
| Protocolo de Investigación Respuesta de los servicios de salud en el abordaje clínico de gestantes con Zika en dos municipios de Colombia 2015-2016 |
| Versión 5 |
| Universidad de los Andes Grupo SEP  20-4-2017 |

**Resumen**

La prestación de los servicios para mujeres gestantes con virus del Zika se enmarca dentro de los lineamientos para prestadores de servicios de salud por parte del Ministerio de Salud y Protección Social que es una adaptación de los lineamientos internacionales dados por la OMS. Objetivo: Evaluar la respuesta institucional frente al acceso y calidad de la prestación de los servicios de salud en el abordaje de gestantes con Zika en los municipios de Villavicencio y Cali durante 2015-2017. Metódos: Estudio de abordaje cualitativo y cuantitativo a desarrollar en dos fases: Fase I. Describir y caracterizar la prestación de servicios de salud. Y fase II. Caracterizar las vivencias de las mujeres gestantes con Zika. Resultados esperados: Documento que de cuenta de la respuesta de los los servicios de salud en el abordaje clínico de gestantes con Zika en dos municipios de Colombia 2015-2016.

**Palabras clave**: Mesh: Zika Virus Infection; health services; Health Care Quality, Access, and Evaluation.

Contenido

[Marco teórico 3](#_gjdgxs)

[Justificación 6](#_1fob9te)

[Cali, Valle del cauca 9](#_3znysh7)

[Villavicencio, Meta 9](#_2et92p0)

[Objetivos 11](#_tyjcwt)

[General 11](#_3dy6vkm)

[Específicos 11](#_1t3h5sf)

[Metodología 12](#_4d34og8)

[Seguridad de datos 17](#_26in1rg)

[Consideraciones éticas y medioambientales 18](#_lnxbz9)

[Principios Éticos Básicos 18](#_35nkun2)

[Difusión de los resultados y publicación 19](#_1ksv4uv)

[Cronograma 19](#_44sinio)

[Presupuesto 19](#_2jxsxqh)

[Equipo de trabajo propuesto 20](#_z337ya)

[Bibliografía 22](#_3j2qqm3)

[Anexos 25](#_1y810tw)

[Anexo 1 25](#_4i7ojhp)

[Anexo 2 28](#_1ci93xb)

# **Marco teórico**

El abordaje de las enfermedades trasmitidas por vectores históricamente ha estado influenciado desde el modelo conocido como de factores de riesgo o canadiense en el cual hay un agente causal y unos factores condicionantes de la salud en la población que se ubican en cuatro dimensiones de la realidad denominadas “La biología humana, el ambiente, los estilos de vida y la organización de los sistemas”(1). A partir de este modelo conceptual, en la región de las Américas se ha desarrollado estrategias de Implementación de la metodología de comunicación para lograr cambios de conducta en los individuos, familias y comunidades con una visión ecosistémica, en la cual el centro son las personas por lo que se hace necesario trabajar sobre los estilos de vida; luego las familias sobre las cuales hay intervenciones educativas y en un círculo más externo está la comunidad entendida como una agregación de vecinos. Bajo el enfoque de riesgo es necesario mejorar los estilos de vida, cambiar hábitos, mejorar los entornos domiciliarios y peri domiciliarios para garantizar agua potable y a la erradicación de criaderos de mosquitos. Las anteriores estrategias pueden ser validas pero insuficientes para la prevención y control de las ETV y hacen que estas se mantengan como ha ocurrido en Colombia en un nivel hiper-endémico.

En Colombia, se realiza la estrategia que se basa en el modelo canadiense de gestión integral dengue, el cual es un modelo de gestión que tiene como objetivo hacer un abordaje integrado entre 6 componentes de la estrategia (vigilancia epidemiológica, atención al paciente, diagnóstico por laboratorio, control integrado del vector, comunicación social y medio ambiente) con vistas a reducir la morbilidad, la mortalidad y la carga social y económica generada por el dengue o en general las ETV, esta misma estrategia se asumió para chikungunya y Zika ya que no se tenía un alistamiento para la epidemia que se generó pero sí una experiencia consolidada en el manejo del dengue, sin embargo frente a la epidemia generada se visibilizan problemáticas que después de aproximados 20 años de haber implementado este modelo no se ha superado, es por esto que para el manejo de las enfermedades transmitidas por vectores se hace necesario una estrategia más integradora, concretamente para Zika se requiere que la estrategia permita la afectación positiva de determinantes sociales y ambientales, lo cual surge frente a las siguientes necesidades y problemas:

- Las ETV como manifestación de no disponibilidad de agua potable y deficiente calidad de vida.
- La capacidad de respuesta local como manifestación de la débil respuesta y acompañamiento nacional.
- Necesidad de Problematizar.

Investigar e intervenir las ETV desde el modelo de Determinantes sociales de la OMS implica un ejercicio de problematización es decir identificar los Determinantes Estructurales entendidos estos como: Contextos sociales y políticos, modelo de desarrollo, modelos de producción y sostenibilidad ambiental en los territorios. Así mismo como Determinante Estructural esta la posición social de las personas y la Salud global. A nivel de Determinantes Intermedios están las condiciones materiales en que viven las personas, su vivienda, servicios de salud y disponibilidad de agua potable.

El modelo teórico inicialmente planteado tiene un abordaje que incluye los determinantes estructurales e intermedios y se grafica de la siguiente manera:

Figura No 1 Modelo teórico Prestación de servicios y ZIKA













Fuente: Elaboración Equipo de Salud Pública Universidad de los Andes 2017

Este modelo permite visibilizar que la prestación de los servicios a las mujeres gestantes se para desde los determinantes intermedios y que de allí se despliegan una serie de factores que no permiten enmarcar la real problemática para el manejo de las enfermedades tropicales.

Actualmente en el país las medidas de control y prevención de esta enfermedad se centran en evitar la picadura del mosquito, reducir la trasmisión sexual y controlar el vector. Entre los potenciales métodos efectivos de prevención centrados en las gestantes se incluye evitar viajar a áreas donde se encuentre reportado la transmisión del virus, evitar el contacto sexual con parejas que se encuentren en riesgo de presentar la infección por el virus del Zika (2) y la utilización de repelente, manejo con permetrina para la ropa (3), mosquiteros, protección en las ventanas y la utilización de aire acondicionado.

Estas medidas se acercan a la población desde las instituciones prestadoras de servicios de salud por medio de conjunto de prestación de servicios referido como el conjunto de derechos, concretados o definidos explícitamente en términos de servicios, tecnologías o pares de estados de necesidad-tratamiento (4). que el sistema sanitario se compromete a proveer a una colectividad. El término prestación se refiere a un beneficio o servicio que una organización suministra a un individuo (4). El conjunto de prestaciones de salud hace referencia, en este documento, al conjunto, lista o relación de servicios o prestaciones financiados total o parcialmente por un sistema público, que son objeto potencial derechos y obligaciones para los actores de dicho sistema (4).

Para la evaluación de las prestaciones de servicios se deben tener en cuenta: 1) los objetivos que motivan su establecimiento, 2) la delimitación de la población beneficiaria, 3) la definición del contenido del conjunto de prestaciones, 4) los criterios de priorización de las prestaciones, 5) la consideración de necesidades diferenciales de grupos concretos, 6) el cálculo del costo del conjunto de prestaciones y 7) la actualización y revisión del contenido del conjunto de prestaciones (4). La prestación cuenta con características de accesibilidad, calidad y eficiencia las cuales corresponden a (4):

Características de la Accesibilidad:

- El que corresponde a la capacidad de los usuarios para buscar y obtener atención
- El que corresponde al tipo y forma de organización de los servicios, para garantizar la atención a los ciudadanos de manera oportuna e integral.
- El de las relaciones que se establecen entre la población, los aseguradores, las entidades territoriales y prestadoras de servicios de salud.

Características de la Calidad:

- La calidad de la atención de salud se entiende como la provisión de servicios accesibles, equitativos, con un nivel profesional óptimo que tiene en cuenta los recursos disponibles y logra la adhesión y satisfacción del usuario.
- El eje de calidad en la política de prestación de servicios se concibe como un elemento estratégico que se basa en dos principios fundamentales: el mejoramiento continuo de la calidad y la atención centrada en el usuario.

Características de la Eficiencia:

- En esencia, el eje de eficiencia se refiere a la obtención de los mayores y mejores resultados, empleando la menor cantidad posible de recursos.
- Si existen dos estrategias de asistencia, igualmente eficaces o efectivas, la menos costosa es la más eficiente

Para la Política Nacional de Prestación de Servicios de Colombia, los aspectos estudiados que generan fallas en la prestación del servicio son:

- **Baja capacidad resolutiva:** Se ha evidenciado baja capacidad resolutiva de las instituciones de baja y media complejidad, con factores asociados a la deficiente actualización del recurso humano, las condiciones de la infraestructura y la forma en que se determina e incorpora la tecnología en estas instituciones
- **Falta del sistema integral de información en salud:** La información sobre prestadores y prestación de servicios de salud es fragmentada y no responde a las necesidades del país. El sistema integral de información en salud y su componente de prestación de servicios está por desarrollarse, situación que limita el direccionamiento, la planeación y la gestión de los prestadores de servicios
- **Debilidad en los procesos de articulación departamento / municipio**: El desarrollo y organización de la red de servicios en las regiones y departamentos, en muchos casos no ha respondido a las necesidades y características de la región, lo que ha generado duplicidad e ineficiencia en la prestación de los servicios. Existen serias limitantes en la capacidad de gestión de las redes de servicios por parte de los departamentos, lo que se suma a las decisiones de municipios que no consultan la realidad local ni regional
- **Debilidades en la gestión.** Existen debilidades de gestión en las entidades de dirección, aseguramiento y prestación de los servicios, tanto públicas como privadas. Las juntas directivas en general, no ejercen el rol de direccionamiento y evaluación, y los gerentes han dejado de lado el tema de la calidad de la gestión clínica y del talento humano
- **Fragmentación y atomización en la prestación de servicios**. La ausencia de articulación y direccionamiento de la oferta, mantiene a los usuarios con servicios fragmentados que limitan el acceso y la integralidad de la atención. En el país, los conceptos de redes de servicios y de modelos de atención en salud son la opción a la fragmentación y atomización en la prestación de servicios

Específicamente para el caso de Zika en el país se estableció que las obligaciones en el marco de la implementación de las acciones para el control del Zika las instituciones prestadoras de salud poseen las siguientes las competencias:

1. Contar con la disponibilidad de consulta pre-concepcional, asesoría y consulta en anticoncepción y métodos de anticoncepción moderna.
2. Realizar un plan de monitoreo al cumplimiento de la difusión de información, a la atención y a la provisión efectiva de métodos de anticoncepción moderna.
3. Tener mecanismos para la entrega de información masiva e interpersonal a mujeres y hombres, sobre la recomendación de postergar el embarazo
4. Acceso a servicios oportunos de consulta pre-concepcional, asesoría y consulta en anticoncepción en el marco de los derechos sexuales y los derechos reproductivos

# **Justificación**

La infección por el virus de Zika a nivel mundial, especialmente en la región de las Américas, ha permitido que los servicios de salud y las políticas públicas en salud de los países afectados ahonden sus esfuerzos en aumentar y mantener la capacidad para detectar y confirmar casos de infección por el virus del Zika, dar tratamiento oportuno a los pacientes, fortalecer las actividades de control a las gestantes y ahondar esfuerzos en reducir el vector transmisor (5). **El principal objetivo de los países** es brindar y asegurar la atención oportuna (interrupción voluntaria del embarazo, prácticas sexuales seguras, atención del parto) a mujeres en edad fértil, gestantes y sus parejas sexuales de conformidad con las lineamientos la legislación y políticas nacionales (6).

El virus del Zika, desconocido para la mayoría de las personas hasta el año 2015, fue identificado por primera vez en Uganda en un mono Rhesus centinela en el año 1947(7). Desde entonces se han descrito brotes esporádicos en algunas regiones del mundo, especialmente en África. El primer informe del virus del Zika fuera de África y Asia fue en el año 2007 cuando el virus se asoció con un brote en el estado de YAP, isla perteneciente de los Estados Federados de Micronesia (8) . Desde el año 2014 y hasta agosto del 2016, según el informe Situacional del Virus Zika de la OMS 169 países o territorios han notificado infecciones por el virus del Zika(6). Entre los países reportados se encuentran los del sudeste asiático; la Polinesia francesa y partes de Sudamérica, Centroamérica, el Caribe y América del Norte (9)(10).

En las Américas se reportó el primer caso de transmisión local del virus en Brasil en el año 2015 y a partir de ese momento más de 45 territorios del continente americano han confirmado casos autóctonos de virus del Zika, convirtiéndose así en un importante problema de Salud pública (11). La OMS/OPS emite en diciembre de 2015 una alerta epidemiológica sobre la posible relación tempero-espacial entre el virus Zika y el incremento en los casos de Síndrome de Guillan Barre y de Anomalías congénitas(6); y en febrero de 2016 declaró la Epidemia del Virus Zika una emergencia de salud pública de carácter internacional debido al alto número de territorios afectados y al alto número de casos de microcefalia (12). Recomienda a los estados miembros aumentar y mantener la capacidad para detectar y confirmar casos de infección por virus Zika, dar tratamiento oportuno a los pacientes, fortalecer las actividades de control a las gestantes y ahondar esfuerzos en reducir el vector transmisor (6).

A partir de la notificación de la epidemia en la región se dieron una serie de estudios que han determinado la relación del virus con las complicaciones de la enfermedad comprobando la presencia del virus en tejidos como la placenta(13–15), líquido amniótico, cerebro, líquido cefalorraquídeo y semen de personas infectadas por el virus Zika (16–18) (13,19,20).

Según datos OMS/OPS durante el 2016, 15 países del continente americano confirmaron casos de síndrome congénitos asociados a la infección por el virus Zika siendo el principal país Brasil con 1.845 casos (98%), seguido por Colombia con 29 reportes y Estados Unidos con 21 casos (6).

De acuerdo a esta situación la OMS lanzo una estrategia conjunta para el tratamiento e identificación de casos de Zika en la gestación y de microcefalia en fetos o neonatos, dentro de la propuesta se identifican recomendaciones a nivel individual y poblacional “Las medidas de protección personal recomendadas incluyen el uso de ropa que cubre todo el cuerpo posible, mosquiteros, pantallas de puertas y ventanas, repelentes seguros y evitar la actividad sexual sin protección con un compañero posiblemente expuesto al virus Zika(21). Además de recomendar medidas locales para reducir los sitios de reproducción de vectores, la orientación señala la importancia de abordar los determinantes sociales subyacentes de este brote y alienta a los gobiernos a adoptar medidas más amplias para proporcionar un acceso sostenible y equitativo al agua potable, al saneamiento ya la gestión apropiada de desechos. La atención prenatal en el contexto de la transmisión del virus Zika requiere acciones específicas para prevenir la infección e identificar a las mujeres que podrían estar infectadas para las pruebas, el cuidado apropiado y el seguimiento”(22,23)

En Colombia las condiciones para la circulación y transmisión del virus Zika son óptimas, ya que los vectores se encuentran distribuidos en las áreas urbanas y rurales de 845 municipios (áreas debajo de los 2200 msnm). Ciudades como Cali en el valle del Cauca y el departamento del Meta registran altos niveles de infestación de formas inmaduras del mosquito Aedes aegypti con índices aedicos de Breteau >5 y < a 50 para el Meta e Índice aédico 3.1 en la ciudad de Cali (24).

Según el boletín epidemiológico a la semana 52 del año 2016 (24) “se han confirmado 6363 casos en mujeres embarazadas y se han notificado 13383 casos sospechosos en gestantes que refieren haber tenido en algún momento síntomas compatibles con enfermedad por virus Zika, de los cuales 12792 casos proceden de municipios donde se confirmó circulación del virus Zika (sospechosos por clínica) y 591 casos de municipios donde no se han confirmado casos”(24).

Respecto a los síndromes neurológicos con antecedentes de enfermedad compatible con infección por virus Zika el INS a la semana 52 reporta desde el “15 de diciembre de 2015 y con corte a la semana epidemiológica 52 de 2016 se han notificado al sistema de vigilancia epidemiológica 677 casos de síndromes neurológicos (Síndrome de Guillain-Barré, polineuropatías ascendentes, entre otras afecciones neurológicas similares) con antecedente de enfermedad febril compatible con infección por virus Zika, los cuales se encuentran en proceso de verificación razón por la cual puede haber ajustes en la notificación acumulada para el evento. Según entidad territorial de residencia la mayor proporción de casos la registró el departamento de Valle del Cauca con 88 casos (el 13 %)”(24).

Conforme a los datos anteriores, en Colombia y toda la región de las Américas las recomendaciones a emitir respecto a postergar o evitar el embarazo, el acceso y uso de métodos de planificación modernos en mujeres y hombres residentes y viajeros a zonas donde circula el virus Zika han sido enfocadas desde el modelo de riesgo, se hace necesario hacer un abordaje desde los determinantes sociales y ambientales de la salud que permita dar una atención integral a las gestantes y mujeres en edad fértil con riesgo de adquirir la enfermedad (21).

En Colombia, 73% de todos los nacimientos ocurren con mujeres de rangos de edad de 15 a 29 años (25). La mayor incidencia de infección por virus del Zika se encuentra en este mismo rango de edad (26). La alta incidencia de infección en este grupo de edad es una preocupación importante y resalta la urgencia en generar esfuerzos en la prevención en este grupo de edad, que pueden tener embarazos intencionados o no intencionados. Además, la asociación ya descrita en alteraciones en el producto del embarazo por infección del virus del Zika enfatiza la necesidad de hacer un monitoreo exhaustivo en las gestantes e hijos que lleguen a presentar la infección.

Para este documento se evaluará la respuesta institucional frente al acceso y calidad de la prestación de los servicios de salud en el abordaje de gestantes con Zika en los municipios de Villavicencio, Meta y Cali, Valle del Cauca durante 2015-2016 en donde se debe tener en cuenta los lineamientos dados por el Instituto Nacional de Salud y el Ministerio de Salud que establecen la notificación obligatoria de los casos sospechosos al sistema de vigilancia SIVIGILA.

# **Cali, Valle del cauca**

Cali es un [municipio](https://es.wikipedia.org/wiki/Municipios_de_Colombia) [colombiano](https://es.wikipedia.org/wiki/Colombia) y es la capital del [departamento](https://es.wikipedia.org/wiki/Departamentos_de_Colombia) del [Valle del Cauc](https://es.wikipedia.org/wiki/Valle_del_Cauca)a; es la [tercera ciudad](https://es.wikipedia.org/wiki/Anexo:Municipios_de_Colombia_por_poblaci%C3%B3n) más poblada de [Colombia](https://es.wikipedia.org/wiki/Colombia). Está situada en la región [Sur del Valle del Cauca](https://es.wikipedia.org/wiki/Sur_del_Valle_del_Cauca), entre la cordillera occidental y la cordillera central de los [Andes](https://es.wikipedia.org/wiki/Regi%C3%B3n_Andina_(Colombia)) (27). La ciudad forma parte del [Área Metropolitana de Cali](https://es.wikipedia.org/wiki/%C3%81rea_Metropolitana_de_Cali), está dividida en 15 corregimientos, 22 comunas y 249 barrios lo que lleva a una superficie total de 564 millones de Kilómetros cuadrados de área de superficie y un total de 2`420.013 habitantes, distribuidos mayormente en el ámbito urbano (27).

En el contexto de la enfermedades por vectores en Colombia, Cali ha aportado en gran número a los casos confirmados en el país por su localización y altitud (24). Desde el año 2013 las enfermedades como el dengue, chikungunya (desde el 2014) y Zika (desde el 2015) han tenido un comportamiento endémico- Epidémico con ciclos de cada 3 a 5 años hasta tener en los últimos 2 años epidemias en cada uno (2015 y 2016) (28).

Específicamente en el tema relacionado con Zika, para el año 2016 se reportaron al SIVIGILA a la semana epidemiológica 52 en la ciudad de Cali 15325 casos que representan el 59% de los casos del departamento, de éstos el 62,2% corresponde a mujeres (9538) (29).

Para el caso de las gestantes, se reportaron 2754 casos sospechosos y confirmados al SIVIGILA en el departamento del Valle de los cuales el 52,5% (1447) mujeres residen en la ciudad de Cali (29), 1439 gestantes ingresaron a estudio por sospecha de Zika. 171 gestantes fueron confirmadas para Zika por PCR en el laboratorio (11.9%) y son objeto de seguimiento para malformaciones, muertes fetales y/o cuadros neurológicos. En 1268 gestantes, el PCR es negativo o no procesado De las 171 gestantes con PCR +, en 8 gestantes (4.7%) ocurrió muerte intrauterina. De las 1268 gestantes con PCR negativo ó no procesado en 23 ocurrieron muertes intrauterinas (1.81%), para un total de 31 muertes intrauterinas de madres con sospecha de Zika (2.15%)(28).

## Villavicencio, Meta

Villavicencio es la capital del departamento de Meta el cual está ubicado en el Piedemonte de la Cordillera Oriental, tiene una población urbana de 450.000 habitantes aproximadamente. El municipio se encuentra dividido en 8 comunas, 235 barrios (de los cuales 32 son legalizados), 101 asentamientos, 2 zonas de invasión, 7 corregimientos y 61 veredas en total (30).

En el departamento del Meta, y específicamente en el municipio de Villavicencio, se han confirmado desde el inicio de la fase epidemia (semana epidemiológica 39 de 2015) hasta la semana epidemiológica 52 de 2016, 2284 casos al SIVIGILA y se han registrado 548 casos de infección por este virus en gestantes cono 14 casos de microcefalia secundaria(31).

En cuanto a las características sociodemográficas de la infección por virus del Zika, se evidenció que el sexo femenino es afectado en una proporción de 2,34:1 respecto al sexo masculino, con una mayor ocurrencia en las cabeceras municipales que en las zonas rurales y, además, los grupos etarios más afectados son los que comprenden de los 20 a los 44 años de edad, que se relaciona con la edad fértil(31). Además, el 42,9% de las gestantes adquirieron el virus del Zika durante el primer trimestre de la gestación, periodo durante el cual se registra el mayor riesgo para el desarrollo de las anomalías congénitas.

# **Objetivos**

# **General**

Evaluar la respuesta institucional frente al acceso y calidad de la prestación de los servicios de salud en el abordaje de gestantes con Zika en los municipios de Villavicencio, Meta y Cali, Valle del Cauca durante 2015-2016.

# **Específicos**

1. Caracterizar la demanda institucional de mujeres gestantes con ZIKA en los dos municipios estudiados.
2. Describir la prestación de servicios de salud a las mujeres gestantes con ZIKA y la atención frente a los síndromes neurológicos y congénitos desde un marco de determinantes sociales.
3. Interpretar a partir de las vivencias de las mujeres gestantes con ZIKA la prestación de los servicios de salud sexual y reproductiva en los municipios estudiados.

# **Metodología**

Figura No 1 Modelo teórico Prestación de servicios y ZIKA














Fuente: Elaboración Equipo de Salud Pública Universidad de los Andes 2017

Teniendo encuenta el modelo teórico propuesto nos concentraremos en los determinantes intermedios, específicamente prestación de servicios de salud. En este sentido el tipo de diseño que se plantea es cuantitativo y cualitativo.

En ese sentido el estudio se dividirá en dos fases metodológicas, buscando dar cumplimiento al os objetivos propuestos:

**Fase I. Caracterizar la demanda institucional de mujeres gestantes con ZIKA en los dos municipios estudiados**: Se realizará una revisión documental que incluye la revisión de fuentes secundarias (boletines epidemiológicos, análisis de situación en salud) del municipio, además se utilizará la información generada a la secretaría de salud municipal. En esta fase se espera obtener los siguientes resultados:

- Número de Instituciones prestadoras de servicios de salud, (IPS) con casos confirmados totales de ZIKA
- Número de Instituciones prestadoras de servicios de salud, (IPS) con casos confirmados por clínica y laboratorio de ZIKA
- Relación de Empresas promotoras de salud e Instituciones prestadoras de Servicios con casos confirmados totales del ZIKA.
- Número de Instituciones prestadoras de servicios de salud, (IPS) con casos confirmados totales de ZIKA relacionados con cada uno de los desenlaces, (IVE, Aborto espontaneo, Microcefalia-Muerto-Nino, Niña Sano)
- Clasificación de IPS según desenlace así:
  - IPS con desenlace IVE
  - IPS con desenlace (Aborto espontaneo, Microcefalia-Muerto-Nino, Niña Sano)
  - IPS con desenlace (**Nino, Niña Sano)**

**Fase II. Caracterizar e Interpretar a partir de las vivencias de las mujeres gestantes con ZIKA la prestación de los servicios de salud sexual y reproductiva en los municipios**: Una vez realizada la clasificación de las IPS de manera conjunta con la Entidad territorial se seleccionará entre tres y cinco IPS por cada clasificación en total **por cada municipio se seleccionará entre 9 y 15 IPS.**

- - IPS con desenlace IVE (tres- **Cinco IPS) por cada municipio**
  - IPS con desenlace (Aborto espontaneo, Microcefalia-Muerto-Nino, Niña Sano) (tres- **Cinco IPS) por cada municipio**
  - IPS con desenlace (**Nino, Niña Sano)** (tres- **Cinco IPS) por cada municipio**

1. **De estas IPS se seleccionará al azar prestadores de servicios de salud y mujeres** a quien se les realizará entrevistas semiestructuradas o grupos focales, dependiendo el caso y la situación; actores claves propuestos:
   - Profesionales de salud responsables del programa de salud sexual y reproductiva de IPS, Empresas promotoras de salud, Secretarías de Salud. Se propone por cada municipio **entre 9 y 12 entrevistados, seleccionados al azar por cada municipio.**
   - Profesionales de salud que prestan servicios de atención en salud esto es consulta médica, consejería, programas de promoción y prevención. Consejería y seguimiento **entre 9 y 12 entrevistados, seleccionados al azar por cada municipio.**
   - Profesionales de salud que están en los servicios de IVE **entre 3 a 5 por cada municipio.**
   - Mujeres con alguno de los desenlaces anteriormente mencionados **entre 10 a 15 por cada municipio**

#### **Categorías de indagación cualitativas**

Revisando los procesos propuestos por la OMS-OPS- adecuados por el Ministerio de Salud y Protección Social de salud y el Instituto Nacional de Salud, de acuerdo al flujograma de atención propuesto en Colombia para la atención, se identificaron cuatro grandes categorías:

- Contexto e Institución
- Aspectos operativos de la atención
- Aspectos técnicos de la atención
- Resultados esperados de la atención

Estas categorías a su vez se estructuran en varias subcategorías como se presenta en las siguientes tablas:

**Tabla 1. Contexto territorial.**

| Fases | Variables |  |  |  |
| --- | --- | --- | --- | --- |
|  |  | **Entrevista responsables del programa salud sexual y reproductiva** | **Entrevista profesionales de salud** | **Entrevista Mujeres** |
| Contexto territorial | Estado de los centros de salud | X | X | X |
|  | Nivel de gestión local | X |  | X |
|  | Condiciones socio culturales de los entornos familiares de las gestantes atendidas |  | X | X |
|  | Barreras de acceso a los servicios de salud | X | X | X |
|  | Contexto socio económico y cultural del municipio-región del área de estudio | X | X | X |

Fuente: Elaboración propia grupo de salud pública Universidad de los Andes.

**Tabla 2. Aspectos operativos de la atención.**

| Fases | Variables |  |  |  |
| --- | --- | --- | --- | --- |
|  |  | **Entrevista responsables del programa salud sexual y reproductiva** | **Entrevista profesionales de salud** | **Entrevista Mujeres** |
| Aspectos Operativos de la atención | Acceso a la información sobre ZIKA | X | X | X |
|  | Tiempo, distancias |  |  | X |
|  | Oportunidad en la atención | X | X | X |
|  | Calidad de la atención | X | X | X |
|  | Integralidad en la atención | X | X | X |
|  | Otros aspectos relevantes | X | X | X |

Fuente: Elaboración propia grupo de salud pública Universidad de los Andes.

**Tabla 3. Aspectos técnicos de la atención.**

| Fases | Variables |  |  |  |
| --- | --- | --- | --- | --- |
|  |  | **Entrevista responsables del programa salud sexual y reproductiva** | **Entrevista profesionales de salud** | **Entrevista Mujeres** |
| Aspectos técnicos de la atención | Implementación de Protocolos | X | X |  |
|  | Conocimiento y manejo de protocolos | X | X | X |
|  | Consejería y seguimiento adecuado de casos | X | X | X |
|  | Manejos claros, concretos | X | X | X |
|  | Otros aspectos relevantes | X | X | X |

Fuente: Elaboración propia grupo de salud pública Universidad de los Andes.

**Tabla 4. Resultados esperados de la atención.**

| Fases | Variables |  |  |  |
| --- | --- | --- | --- | --- |
|  |  | **Entrevista responsables del programa salud sexual y reproductiva** | **Entrevista profesionales de salud** | **Entrevista Mujeres** |
| Resultados esperados dela atención | Tiene en cuenta los derechos sexuales y reproductivos delas mujeres | X | X | X |
|  | La ruta seguida fue concertada de acuerdo a las necesidades y situación clínica de la mujer |  | X | X |
|  | Los tratamientos y recomendaciones ordenados fueron oportunamente suministrados bajo un enfoque de derechos. | X | X | X |
|  | Otros aspectos relevantes | X | X | X |

Fuente: Elaboración propia grupo de salud pública Universidad de los Andes.

#### **Instrumentos de recolección cualitativos**

Los instrumentos utilizados para la recolección de información en el componente cualitativo de la evaluación se pueden observar en el anexo 1. A continuación, se hace una breve descripción de los instrumentos utilizados:

**Entrevista semi estructurada** La entrevista es fundamentalmente una conversación o diálogo sistematizado, cuyo objeto es lograr, recobrar y registrar las experiencias de vida de las personas en torno a un hecho particular. El propósito de semi estructurar es permitir al interlocutor una mayor libertad de expresión de su vivencia.

# **Plan de análisis de la información**

1. **Análisis cualitativo**

- Transcripción de entrevistas relevantes y del CSC
- Reunión de investigadores que estuvieron en campo para hacer un análisis del mismo, desde los diarios de campo.
- Una vez se tengan las transcripciones de las entrevistas y la revisión de los contextos se procederá a hacer un análisis interpretativo de contenido de los mismos
- Para el análisis de la información recolectada en el CSC se recurrirá a un análisis del discurso.
- Triangulación de la información obtenida.
- Escritura del documento final.

El manejo de la información del componente cualitativo será almacenado bajo las mismas condiciones de la información cuantitativa, específicamente los audios generados serán entregados a OPS/OMS. La sistematización y análisis de datos para el componente cuantitativo se realizará utilizando SPSSS y para el componente cualitativo se propone un análisis de la información.

# **Garantía de la calidad del estudio**

El desarrollo del proyecto para caracterizar la respuesta de los servicios de salud en el abordaje clínico de gestantes con Zika en los municipios de Villavicencio y Cali 2015-2017 contempla un equipo de trabajo conformado por el equipo de la Universidad de los Andes-Facultad de Medicina, las Secretarias de Salud Municipales y Departamentales de los lugares del estudio y mujeres de los territorios seleccionados.

Por otra parte, la estructura organizacional de la Universidad de los Andes estará liderada por una directora general de proyecto quien se encargará de supervisar la ejecución del contrato de forma transversal, es decir, desde sus aspectos técnicos y administrativos. A su vez, se contempla contar con líderes de coordinación para el cumplimiento del cronograma, recolección de datos, fechas para entregables y cumplimiento de los objetivos.

# **Seguridad de datos**

Todos los datos se manejarán de forma anónima. Esto incluye el uso de un código en números en lugar de los nombres completos de las transcripciones de las entrevistas y la desvinculación de datos espaciales que podrían ser utilizados para localizar a los sujetos. Los datos fotográficos recogidos serán asegurados en un archivador cerrado con llave. Las fotografías de personas o lugares que podrían presentar un riesgo para los sujetos (u otras personas) serán destruidos.

Se debe proteger la confidencialidad mediante el uso de la protección de contraseña en cualquier equipo que contiene materiales de investigación. Todos los registros de audio y video, transcripciones de entrevistas y notas de campo serán asegurados en un archivador cerrado accesible sólo para el investigador.

Todos los datos y la información de investigación se dispondrán durante un periodo de tres años después de terminar el estudio (para permitir el análisis de datos y la publicación de los resultados). Después de este período de tiempo, los datos (incluidos los datos fotográficos, audios, videos, datos de entrevistas y transcripciones, notas de campo, y todos los demás datos recogidos) serán eliminados.

# **Consideraciones éticas y medioambientales**

Ésta investigación fue sometida al comité de ética de la Universidad de los Andes el cual aprobó como riesgo mínimo la investigación mediante el acta 658 de 2016, esta investigación se realizará respetando los lineamientos internacionales para la investigación en seres humanos estipulados en la Declaración de Helsinki de la Asociación Médica Mundial, el Reporte Belmont, las Pautas éticas CIOMS, La declaración de Budapest sobre la ciencia y el uso del saber científico, así como también la normativa vigente establecida en la Resolución 008430 de Octubre 4 de 1993 de La República de Colombia y la Resolución 2378 de 2008 de la República de Colombia “Por la cual se adoptan las Buenas Prácticas Clínicas para las instituciones que conducen investigación en seres humanos”.

# **Principios Éticos Básicos**

Respeto a las Personas: Respeto por la autonomía; valorar las opiniones y elecciones de hacen las personas con capacidad de autodeterminación. Las personas objeto de interés participaran en la investigación si está es compatible con sus principios, interés y preferencias. Este principio se cumple con el diligenciamiento del consentimiento informado.

El ingreso a este estudio es voluntario y se debe contar con el consentimiento informado (ver anexo No. de la persona el manejo prestado a las gestantes con exposición al virus del Zika, caracterizar la atención recibida los aciertos y fallas el abordaje integral de las gestantes, sus hijos y su comunidad)

#

# **Difusión de los resultados y publicación**

Se realizarán dos publicaciones en revistas indexadas, una nacional y otra internacional. Se realizará la exposición de los resultados en dos congresos nacionales de salud pública.

# **Cronograma**

| Fases | Actividad / producto final | Meses | | | | | | | | | | | |
| --- | --- | --- | --- | --- | --- | --- | --- | --- | --- | --- | --- | --- | --- |
|  |  | 1 | 2 | 3 | 4 | 5 | 6 | 7 | 8 | 9 | 10 | 11 | 12 |
| **Fase I. Descripción y caracterización de la prestación de los servicios** | Documento en el que se describa cómo se da la prestación de los servicios y los diferenciales en público y privado. |  |  |  |  |  |  |  |  |  |  |  |  |
| **Fase II.** **Caracterizar las vivencias de las mujeres gestantes con Zika** | Sistematización de los dos conversatorios, uno en cada municipio, y de las entrevistas semiestructuradas realizadas. |  |  |  |  |  |  |  |  |  |  |  |  |
| **Fase IV. Análisis de los resultados** | Entrega de dos artículos para publicación uno nacional y uno internacional. |  |  |  |  |  |  |  |  |  |  |  |  |

# **Presupuesto**

Ver anexo.

# **Equipo de trabajo propuesto**

| Nombre y apellidos | Sexo  (M / H) | Nombre de la institución | Competencia técnica | Función en el proyecto | EJC (%) |
| --- | --- | --- | --- | --- | --- |
| JOVANA OCAMPO CAÑAS | M | UNIVERSIDAD DE LOS ANDES | PhD en Salud Publica: Diseño, ejecución, revisión de investigación. Análisis de Datos. Interpretación de los determinantes sociales de la salud. | Investigador Principal | 40% |
| SOFIA RIOS | M | UNIVERSIDAD DE LOS ANDES | Magister en Salud Publica: Diseño, ejecución, revisión de investigación. Análisis de Datos. | Co investigador | 30% |
| YANETH PINILLA | M | UNIVERSIDAD DE LOS ANDES | Antropóloga Experta investigadora Cualitativa | Co investigadora | 40% |
| LUIS JORGE HERNÁNDEZ | H | UNIVERSIDAD DE LOS ANDES | PhD en Salud Publica: Diseño, ejecución, revisión de investigación. Análisis de Datos. Interpretación de los determinantes sociales de la salud. | Co investigador | 15% |
| LUIS ANDRES SARMIENTO | H | UNIVERSIDAD DE LOS ANDES | Especialista en Ginecobstetricia; investigador en IVE Diseño, ejecución, revisión de investigación. Análisis de Datos. | Co investigador | 10% |
| ALEXANDRA PARDO SALAZAR | M | Secretaria de salud del Departamento del Meta | Especialista en Epidemiologia: Diseño, ejecución, revisión de investigación. Análisis de Datos. | Co investigador | 40% |
| JAVIER COLORADO | H | Secretaria de Salud Municipal de Cali | Médico, Diseño, ejecución, revisión de investigación. Análisis de Datos. | Co investigador | 20% |
| CLAUDIA DEL PILAR CALDERON | M | UNIVERSIDAD DE LOS ANDES | Médica, Maestría en Enfermedades de Trópico. Diseño, ejecución, revisión de investigación. Análisis de Datos | Co investigador | 20% |
| ANDRES MAURICIO GARCIA | H | Estudiante de Maestría de Salud Pública UNIVERSIDAD DE LOS ANDES | Diseño, ejecución, revisión de investigación. Análisis de Datos. | Co investigador | 30% |
| HECTOR MANUEL GOMEZ | H | Estudiante de Maestría de Salud Pública UNIVERSIDAD DE LOS ANDES | Diseño, ejecución, revisión de investigación. Análisis de Datos. | Co investigador | 40% |
| LUIS GUILLERMO GÓMEZ | H | Estudiante de Medicina UNIVERSIDAD DE LOS ANDES | Diseño, ejecución, revisión de investigación. Análisis de Datos. | Co investigador | 20% |
| ANDRES FIDEL MORENO | H | Estudiante de Medicina UNIVERSIDAD DE LOS ANDES | Diseño, ejecución, revisión de investigación. Análisis de Datos. | Co investigador | 20% |
| JULIANA ZAMBRANO | M | Estudiante de Medicina UNIVERSIDAD DE LOS ANDES | Diseño, ejecución, revisión de investigación. Análisis de Datos. | Co investigador | 20% |

# **Bibliografía**

1. Lip C, Rocabado F. Determinantes sociales de la salud en Perú. Cuadernos de Promoción de la Salud. 2005. 84 p.

2. OMS. Atención en el embarazo en el contexto del brote de virus de Zika Orientación provisional actualizada [Internet]. Ginebra : OMS ; 2016 [cited 2017 Mar 9]. p. 1–15. Available from: http://apps.who.int/iris/bitstream/10665/204617/1/WHO_ZIKV_MOC_16.2_spa.pdf?ua=1

3. Banks SD, Murray N, Wilder-Smith A, Logan JG. Insecticide-treated clothes for the control of vector-borne diseases : a review on effectiveness and safety. Med Vet Entomol. 2014;28(Suppl. I):14–25.

4. OPS/OMS. Conjuntos de prestaciones de salud [Internet]. 2003. 154 p. Available from: http://www.paho.org/hq/documents/conjuntosdeprestacionesdesaludobjetivosdisenoyaplicacion-ES.pdf

5. Petersen LR, Jamieson DJ, Powers AM, Honein MA. Zika Virus. N Engl J Med [Internet]. 2016;374(16):1552–63. Available from: http://www.nejm.org/doi/10.1056/NEJMra1602113%5Cnhttp://www.ncbi.nlm.nih.gov/pubmed/27028561

6. OPS/OMS. Alerta Epidemiológica Síndrome neurológico, anomalías congénitas e infección por virus Zika. Implicaciones para la salud pública en las Américas. 1 de diciembre de 2015. (Organización Panam la Salud y Organ Mund la Salud). 2015;1–12.

7. Wikan N, Smith DR. Zika virus: History of a newly emerging arbovirus. Vol. 16, The Lancet Infectious Diseases. 2016. p. e119–26. 29

8. Duffy M. Chen T.Hancock T. Powers A. Kool J. Lanciotti R. Pretrick M. Zika Virus Outbreak on Yap Island, Federated States of Micronesia. N Engl J Med. 2009;360:2536–43.

9. Messina JP, Kraemer MU, Brady OJ, Pigott DM, Shearer FM, Weiss DJ, et al. Mapping global environmental suitability for Zika virus. Elife [Internet]. 2016;5:e15272. Available from: https://elifesciences.org/content/5/e15272

10. Plourde AR, Bloch EM. A literature review of zika virus. Vol. 22, Emerging Infectious Diseases. 2016. p. 1185–92.

11. Centers for Disease Control. Zika Virus. Centers Dis Control Prev Zika Virus Home [Internet]. 2016;1–12. Available from: http://www.cdc.gov/zika/index.html

12. Rodriguez LC. Microcephaly and Zika virus infection. Lancet [Internet]. 2016;387(10033):2070–2. Available from: http://www.ncbi.nlm.nih.gov/pubmed/26993883

13. Musso D, Roche C, Robin E, Nhan T, Teissier A, Cao-Lormeau VM. Potential sexual transmission of zika virus. Emerg Infect Dis. 2015;21(2):359–61.

14. Calvet G, Aguiar RS, Melo ASO, Sampaio SA, de Filippis I, Fabri A, et al. Detection and sequencing of Zika virus from amniotic fluid of fetuses with microcephaly in Brazil: a case study. Lancet Infect Dis. 2016;16(6):653–60.

15. Barton MA, Salvadori MI. Zika virus and microcephaly. Vol. 188, CMAJ. 2016.

16. Hills MAJLMJRSMGS. Zika and the Risk of Microcephaly. N Engl J Med. 2016;363(1):1–3.

17. Kleber Giovanni, Luz; Glauco Igor Viana dos, Santos; Renata de Magalhães V-S more at: http://search. scielo. org/?q=zika&where=ORG#sthash. t5TdpXrp. dpu., Gewin V, Lancet T, Holden P, Local D, Committee M, et al. Zika Virus Infection in Pregnant Women in Rio de Janeiro — Preliminary Report. Lancet Infect Dis. 2016;6736(16):2016.

18. França GVA, Schuler-Faccini L, Oliveira WK, Henriques CMP, Carmo EH, Pedi VD, et al. Congenital Zika virus syndrome in Brazil: a case series of the first 1501 livebirths with complete investigation. Lancet (London, England). 2016;388(10047):891–7.

19. Besnard M, Lastère S, Teissier A, Cao-Lormeau VM, Musso D. Evidence of perinatal transmission of zika virus, French Polynesia, December 2013 and February 2014. Eurosurveillance. 2014;19(13).

20. Nunes ML, Carlini CR, Marinowic D, Neto FK, Fiori HH, Scotta MC, et al. Microcephaly and Zika virus: A clinical and epidemiological analysis of the current outbreak in Brazil. Vol. 92, Jornal de Pediatria. 2016. p. 230–40.

21. Ministerio de Salud y Protección Social - Dirección de Promoción y Prevención Grupo de Curso de Vida. Lineamientos para la detección y manejo clínico integral de anomalías congénitas en fetos expuestos al virus del Zika durante la gestación de pacientes en colombia ministerio de salud y protección social [Internet]. Bogotá: Ministerio de Salud Y protección Social; 2016 [cited 2017 Mar 9]. Available from: https://www.minsalud.gov.co/sites/rid/Lists/BibliotecaDigital/RIDE/VS/PP/ET/linea-deteccion-manejo-clinico-anomalia-congenitas-fotos-zika.pdf

22. Oduyebo T, Petersen EE, Rasmussen SA, Mead PS, Meaney-Delman D, Renquist CM, et al. Update: Interim Guidelines for Health Care Providers Caring for Pregnant Women and Women of Reproductive Age with Possible Zika Virus Exposure - United States, 2016. MMWR Morb Mortal Wkly Rep [Internet]. 2016;65(5):122–7. Available from: http://www.ncbi.nlm.nih.gov/pubmed/26866840

23. Oladapo OT, Souza JP, De Mucio B, de Le??n RGP, Perea W, G??lmezoglu AM. WHO interim guidance on pregnancy management in the context of Zika virus infection. Vol. 4, The Lancet Global Health. 2016. p. e510–1.

24. Instituto Nacional de Salud. Boletín epidemiológico semanal. Bogotá Colombia; 2016. p. 121.

25. DANE. Nacimientos 2016 [Internet]. Todos por un nuevo país . 2016 [cited 2017 Mar 9]. Available from: http://www.dane.gov.co/index.php/estadisticas-por-tema/salud/nacimientos-y-defunciones/nacimientos/nacimientos-2016

26. Pacheco O, Beltrán M, Nelson C a, Valencia D, Tolosa N, Farr SL, et al. Zika Virus Disease in Colombia - Preliminary Report. N Engl J Med [Internet]. 2016;1–10. Available from: http://www.ncbi.nlm.nih.gov/pubmed/27305043

27. Grupo de vigilancia y eventos transmitidos por vectores. Boletín epidemiológico No. 11 Semana epidemiológica No. 44. Cali: Secretaria de Salud Cali; 2016. p. 26.

28. Municipal SDS. BOLETÍN EPIDEMIOLÓGICO No. 19 SEMANA EPIDEMIOLOGICA No. 52. 2016;(11).

29. Gobernación Valle del Cauca. Boletín Epidemiológico Semanal, Semana epidemiológica 52 de 2016. Vol. 2016. 2016.

30. Secretaria municipal. Villavicencio unidos podemos [Internet]. 28 de marzo. 2017. Available from: http://www.villavicencio.gov.co/index.php?option=com_content&view=article&id=32&Itemid=87

31. Secretaria de Salud de Villavicencio. Comportamiento epidemiológico de los eventos de interés en salud pública, Villavicencio 2016. 2016 p. 1–111.

# **Anexos**

# **Anexo 1**

# **Instrumentos recolección de datos cualitativo.**

| Respuesta de los servicios de salud en el abordaje clínico de gestantes con Zika en dos municipios de Colombia 2015-2016 | | | | | |
| --- | --- | --- | --- | --- | --- |
| GUÍA DE ENTREVISTA Y CONVERSATORIOS A USUARIOS DE SERVICIO DE SALUD | | | | | |
| DATOS DE CONTROL | | | | | |
| ENTREVISTADOR: | | | LUGAR: | FECHA: | HORA DE ENTREVISTA: |
| DEPARTAMENTO: | | | MUNICIPIO | BARRIO O LOCALIDAD: | |
| INFORMACION GENERAL DEL ENTREVISTADO | | | | | |
| NOMBRE: | | | | PARENTESCO: | |
| EDAD: | ETNIA (SI APLICA): | | | OCUPACIÓN: | |
| NUMERO DE PERSONAS QUE COMPONEN HOGAR: | | | | ULTIMO GRADO CURSADO: | |
| REGIMEN DE AFILIACIÓN: | | | | TELEFONO DE CONTACTO: | |
|  | | | | | |
| ENTORNO FAMILIAR | | - ¿En este momento quienes conforman su familia? - Hablemos de su familia, quienes la conforman - Hablemos de sus embarazos - Hablemos en especial de su último embarazo | | | |
|  | | | | | |
| SABERES PROPIOS | | - ¿Cómo cuido de sus embarazos anteriores? - ¿Planeo este último embarazo? - ¿Cómo se enteró que estaba embarazada? - ¿Cómo se cuidó en sus anteriores embarazos? ¿Cómo se cuidó en este embarazo? | | | |
|  | |  | | | |
| SALUD | | **Promoción de la salud de la Gestante** | | | |
|  |  | - ¿Qué debe hacerse para que una gestante se encuentre saludable? - ¿Qué se le debe enseñar a una gestante para que lleve una vida sana y saludable? - ¿Ha recibido algún tipo de capacitación en temas de salud? - ¿Quién o cuál institución en el municipio promociona un estilo de vida saludable en la gestante? | | | |
|  |  | **Prevención** | | | |
|  |  | - ¿Usted asistió a controles Prenatales? - ¿Cuáles cree que son las enfermedades que más afectan a las gestantes? - ¿Conocía o Conoce usted sobre el ZIKA? - ¿Conoce sobre las complicaciones del ZIKA durante la gestación? | | | |
|  | | **Atención** | | | |
|  |  | - ¿Cómo se dio cuenta que tenía ZIKA? - ¿En qué mes de su gestación se dio cuenta? - ¿Cuándo inicio sus controles prenatales? - ¿Qué sintió cuando le sospecharon ZIKA? - ¿Cuándo consulto que le dijeron los médicos o los profesionales de salud sobre su enfermedad durante el embarazo? - Los profesionales de salud respetaron sus creencias y sus derechos sexuales reproductivos - Sabe que son los derechos sexuales reproductivos - Como se siente frente a los resultados de ese proceso de atención cuando le diagnosticaron ZIKA CUANDO ESTABA EMBARAZADA | | | |

| Respuesta de los servicios de salud en el abordaje clínico de gestantes con Zika en dos municipios de Colombia 2015-2016 | | | | | |  |
| --- | --- | --- | --- | --- | --- | --- |
|  | | | | | |  |
|  | | | | | |  |
| GUÍA DE ENTREVISTA A REFERENTES INSTITUCIONALES LOCALES | | | | | |  |
| DATOS DE CONTROL | | | | | | |
| ENTREVISTADOR: | | | LUGAR: | FECHA: | HORA DE ENTREVISTA: | |
| DEPARTAMENTO: | | | MUNICIPIO | BARRIO O LOCALIDAD: | | |
| INFORMACION GENERAL DEL ENTREVISTADO | | | | | | |
| NOMBRE: | | | | PROFESIÓN: | | |
| EDAD: | ETNIA (SI APLICA): | | | OCUPACIÓN: | | |
| LUGAR DONDE TRABAJA: | | | | TELEFONO DE CONTACTO: | | |
|  | |  | | | |  |
| ACERCA DEL TERRITORIO | | - ¿Nos podría mencionar las mayores dificultades para el sector salud de trabajo en la región? - ¿Nos podría mencionar los logros de los últimos años para el sector salud en esta región? - ¿Nos podría mencionar las acciones que se han venido desarrollando en el programa de salud sexual y reproductiva? - ¿Cuál es el perfil epidemiológico relacionado con ZIKA y Gestante para su Municipio en el 2015 y 2016? - ¿Cuáles son las prioridades desde el sector salud para garantizar los derechos sexuales y en la Región? - ¿Y cuáles con las mayores dificultades que han encontrado en el camino? - ¿Cuáles son las prioridades desde el sector salud para garantizar que no haya complicaciones por ZIKA EN GESTANTES en la Región? - ¿Y cuáles con las mayores dificultades que han encontrado en el camino? | | | |  |
|  | |  | | | |  |
| PROTOCOLO DE ZIKA | | - ¿Qué es ZIKA para usted? - ¿qué papel juega la salud sexual y reproductiva en la epidemia del zika especialmente en gestantes? - ¿Nos podría comentar brevemente cómo ha sido el desarrollo y la implementación de los protocolos de manejo de ZIKA en la región? | | | |  |
|  | |  | | | |  |
| ACERCA DE LA OPERACIÓN | | - ¿Quién lideró el proceso de implementación, operación y seguimiento de protocolos de zika en el departamento/municipio? - ¿Cuántos profesionales se lograron capacitar? ¿Dónde? ¿Quién realizó la gestión? - ¿Sienten que los profesionales capacitados han mejorados sus habilidades sobre el manejo de ZIKA en gestantes? - ¿Cuáles aspectos mejorarían? | | | |  |

| Respuesta de los servicios de salud en el abordaje clínico de gestantes con Zika en dos municipios de Colombia 2015-2016 | | | | | |
| --- | --- | --- | --- | --- | --- |
| GUÍA DE ENTREVISTA Y CONVERSATORIO A PRESTADORES DEL SERVICIO DE SALUD | | | | | |
| DATOS DE CONTROL | | | | | |
| ENTREVISTADOR: | | | LUGAR: | FECHA: | HORA DE ENTREVISTA: |
| DEPARTAMENTO: | | | MUNICIPIO | BARRIO O LOCALIDAD: | |
| INFORMACION GENERAL DEL ENTREVISTADO | | | | | |
| NOMBRE: | | | | PROFESIÓN: | |
| EDAD: | ETNIA (SI APLICA): | | | OCUPACIÓN: | |
| LUGAR DONDE TRABAJA: | | | | TELEFONO DE CONTACTO: | |
|  | |  | | | |
| ACERCA DEL TERRITORIO | | - ¿Nos podría mencionar las mayores dificultades para el sector salud de trabajo en la región? - ¿Nos podría mencionar los logros de los últimos años para el sector salud en esta región? - ¿Nos podría mencionar las acciones que se han venido desarrollando en el programa de salud sexual y reproductiva? - ¿Cuál es el perfil epidemiológico relacionado con ZIKA y Gestante para su Municipio en el 2015 y 2016? - ¿Cuáles son las prioridades desde el sector salud para garantizar los derechos sexuales y en la Región? - ¿Y cuáles con las mayores dificultades que han encontrado en el camino? - ¿Cuáles son las prioridades desde el sector salud para garantizar que no haya complicaciones por ZIKA EN GESTANTES en la Región? - ¿Y cuáles con las mayores dificultades que han encontrado en el camino? | | | |
|  | |  | | | |
| PROTOCOLO DE ZIKA | | - ¿Qué es ZIKA para usted? - ¿qué papel juega la salud sexual y reproductiva en la epidemia del zika especialmente en gestantes? - ¿Nos podría comentar brevemente cómo ha sido el desarrollo y la implementación de los protocolos de manejo de ZIKA en la región? | | | |
|  | |  | | | |
| ASPECTOS TÉCNICOS | | - ¿Qué mejoraría de la ruta de atención en Zika para las gestantes? - ¿Cómo definiría brindar un servicio integral a sus pacientes con ZIKA en gestación? - Los protocolos de ZIKA fue o es una herramienta óptima para la atención integral de pacientes con ZIKA | | | |
|  | |  | | | |
| RESULTADOS ESPERADOS | | - ¿Usted considera que dio respuesta efectiva a las pacientes con ZIKA que se encontraban embarazadas? - ¿Qué dificultades se presentaron? - Que no repetiría de este proceso? | | | |
|  | |  | | | |

# **Anexo 2**

# **Consentimiento Informado**

Consentimiento Informado e Información sobre el estudio

“Respuesta de los servicios de salud en el abordaje clínico de gestantes con Zika en dos municipios de Colombia 2015-2016”

Introducción de investigador(a):

Buen día, mi nombre es ____________ y soy ________________.

El objetivo de su participación en el estudio es ayudar al mejoramiento de los servicios de salud en cuanto a medidas de anticoncepción, interrupción voluntaria del embarazo y el abordaje clínico de las gestantes con Zika.

Descripción de Actividades:

Si usted acepta participar en este estudio indica que hará parte como participante del proyecto Respuesta de los servicios de salud frente a los conocimientos, actitudes y prácticas en relación con los métodos anticonceptivos, los servicios de interrupción voluntaria del embarazo y el abordaje clínico de gestantes con zika en los municipios de Villavicencio y Cali 2015-2017.

Beneficios:

Usted no recibirá un beneficio económico ni material por la participación en este estudio. El beneficio será la satisfacción de conocer y entender los indicadores que afectan de manera directa o indirecta la respuesta en salud a las maternas con Zika.

Confidencialidad:

Toda la información que usted comparta en este estudio es confidencial. En ningún momento se revelará su nombre o identidad, así como tampoco se revelarán los datos individuales, pues éstos serán usados solamente por el equipo y serán usados de manera anónima en escritos académicos.

Riesgos Potenciales/Compensación:

Los riesgos por participar en esta investigación son mínimos o nulos y usted no recibirá ningún pago económico como tampoco tendrá costo alguno para usted por participar en este estudio.

Participación Voluntaria:

La participación en este estudio es absolutamente voluntaria. Usted está en plena libertad de negarse a participar. Su decisión no participar no afectará de ninguna manera los beneficios que obtiene de la IPS en la que recibe la atención.

Datos de contacto:

Si tiene alguna pregunta durante o después de que termine el estudio, usted puede llamar a la Dra. Jovana Ocampo al teléfono 3394949 o escribir al correo electrónico [ja.ocampo@uniandes.edu.co](mailto:ja.ocampo@uniandes.edu.co).

Si usted tiene alguna pregunta durante o después de que termine el estudio favor comunicarse con Jovana Ocampo Cañas, su teléfono es 3394949 Ext 1460, su dirección es Cra 1 Nº 18A- 12, facultad de Medicina, piso octavo. En caso de presentarse algún problema asociado a la investigación, usted también puede contactar al Comité de Ética de la Universidad de los Andes. Teléfono 3394949 Ext. 3867 o al correo electrónico [comite-etica-investigaciones@uniandes.edu.co](mailto:comite-etica-investigaciones@uniandes.edu.co)

¿Quisiera usted participar en el estudio? Sí _____ No______

¿Podemos grabar las actividades en audio y/o video? Sí _____ No______

(En caso afirmativo, usted participará en la selección del material audiovisual)

¿Podemos publicar fotografías en las que usted aparezca,

y otra información en el producto académico? Sí _____ No______

__________________________ ____________

Firma del Participante Fecha

_________________________ ____________

Firma del Testigo 1 Fecha

__________________________ ____________

Firma del Testigo 2 Fecha
