# Supplementary material for: Experiences of women with Zika virus (ZIKV) versus the provision of health services in two cities in Colombia: A qualitative study
Source: PLoS One. 2021 Dec 2;16(12):e0260583. doi: 10.1371/journal.pone.0260583 (PMC8638867; doi:10.1371/journal.pone.0260583)
Supplement: Attachment — Submitted filename: PONE-D-20-38136 cpg 7.1.2021 Colombia.pdf [file pone.0260583.s006.pdf]

PLOS ONE

Experiences of women with ZIKV virus (ZIKV) versus the provision of health services in two cities in Colombia: a qualitative study

--Manuscript Draft--

Manuscript Number:	PONE-D-20-38136
Article Type:	Research Article
Full Title:	Experiences of women with ZIKV virus (ZIKV) versus the provision of health services in two cities in Colombia: a qualitative study
Short Title:	Experiences of women with ZIKV virus in two cities in Colombia: a qualitative study
Corresponding Author:	Jovana Alexandra Ocampo Cañas, Ph.D.<br>Universidad de Los Andes<br>Bogotá D.C., COLOMBIA
Keywords:	Experiences; ZIKA Virus; Women Health; Qualitative Study; Colombia
Abstract:	Background: In February 2016, the World Health Organization (WHO) declared the epidemic of the ZIKA virus (ZIKV) in Latin America to be a public health emergency. In Colombia, 11,944 pregnant women registered a ZIKV infection during the epidemic. So far, little is known about the experiences of women infected with ZIKV during their pregnancy, especially those relating to the provision of health services during the period of the epidemic.<br>Objective: To explore the experiences of pregnant women diagnosed with ZIKV infection about the provision of health services in two Colombian cities, considering the perspective of sexual and reproductive rights.<br>Methods: Qualitative study under the grounded theory approach, which uses semi-structured interviews as tools to explore the biographical experience of mothers during their gestation process and ZIKV infection, dividing the interview into two broad categories: before and during pregnancy.<br>Results: Twenty-two women were interviewed, 10 in the city of Cali and 12 in the city of Villavicencio. Information related to health service delivery during the ZIKV epidemic was obtained. In addition, we analyzed data on the transformation of their pregnancy experience when they were infected with ZIKV. Data were also obtained on the assimilation of the news received during pregnancy, the impact of the products of gestation on their lives and in the dynamic of their family, and, lastly, the social and cultural aspects, which determined the level of autonomy of women in the exercise of their sexual and reproductive rights, beyond the context of the epidemic.<br>Conclusions: In the health care of ZIKV epidemics, it is necessary to include the gender perspective, more specifically, sexual and reproductive rights. In addition, these epidemics must be addressed through a comprehensive, appropriate, and not fragmented health system, in which sexual and reproductive rights must be mainstreamed in all health promotion and prevention programs.
Order of Authors:	Jovana Alexandra Ocampo Cañas, Ph.D.
	María Yaneth Pinilla
	Clemencia Del Pilar Navarro Plazas
	Carlos Mauricio Mejía Arbeláez
	Jhon Sebastián Patiño Rueda
Additional Information:	
Question	Response
**Financial Disclosure**<br><br>Enter a financial disclosure statement that describes the sources of funding for the work included in this submission. Review	This work was funded by the HRP Alliance, part of the UNDP/UNFPA/UNICEF/WHO/World Bank Special Programme of Research, Development and Research Training in Human Reproduction (HRP) and the UNICEF/UNDP/World Bank/WHO Special Programme for Research and Training in Tropical Diseases (TDR), both cosponsored programs hosted by the World

Powered by Editorial Manager® and ProduXion Manager® from Aries Systems Corporation

the submission guidelines for detailed requirements. View published research articles from *PLOS ONE* for specific examples.<br><br>This statement is required for submission and **will appear in the published article** if the submission is accepted. Please make sure it is accurate.<br><br>**Unfunded studies**<br>Enter: *The author(s) received no specific funding for this work.*<br><br>**Funded studies**<br>Enter a statement with the following details:<br>• Initials of the authors who received each award<br>• Grant numbers awarded to each author<br>• The full name of each funder<br>• URL of each funder website<br>• Did the sponsors or funders play any role in the study design, data collection and analysis, decision to publish, or preparation of the manuscript?<br>• **NO** - Include this sentence at the end of your statement: *The funders had no role in study design, data collection and analysis, decision to publish, or preparation of the manuscript.*<br>• **YES** - Specify the role(s) played.<br><br>* typeset	HealthOrganization (WHO).<br><br>https://www.who.int/reproductivehealth/hrp_alliance/en/<br><br>The funders had no role in study design, data collection and analysis, decision to publish, or preparation of the manuscript.
**Competing Interests**<br><br>Use the instructions below to enter a competing interest statement for this submission. On behalf of all authors, disclose any competing interests that could be perceived to bias this work—acknowledging all financial support and any other relevant financial or non-financial competing interests.<br><br>This statement **will appear in the published article** if the submission is accepted. Please make sure it is accurate. View published research articles from *PLOS ONE* for specific examples.	The authors have declared that no competing interests exist.

Powered by Editorial Manager® and ProduXion Manager® from Aries Systems Corporation

**NO authors have competing interests**<br><br>Enter: *The authors have declared that no competing interests exist.*<br><br>**Authors with competing interests**<br><br>Enter competing interest details beginning with this statement:<br><br>*I have read the journal's policy and the authors of this manuscript have the following competing interests: [insert competing interests here]*<br><br>* typeset	
**Ethics Statement**<br><br>Enter an ethics statement for this submission. This statement is required if the study involved:<br><br>• Human participants<br>• Human specimens or tissue<br>• Vertebrate animals or cephalopods<br>• Vertebrate embryos or tissues<br>• Field research<br><br>Write "N/A" if the submission does not require an ethics statement.<br><br>General guidance is provided below. Consult the submission guidelines for detailed instructions. **Make sure that all information entered here is included in the Methods section of the manuscript.**	The investigation was carried out under the criteria of resolution 008430 of October 4, 1993 of the Republic of Colombia [18], according to which it is characterized as "without risk" by being immersed in the category of studies using retrospective documentary research techniques and methods and, where no intentional intervention or modification is performed of the biological, physiological, or psychological or social variables of the individuals. Similarly, respect for human dignity was maintained and informed consent was obtained, in which aspects such as the objective, methodology, handling of the information provided, results, disclosure of the findings and the right to reply or not to questions and suspend the interviews when the participants so wish was observed. The protocol of the study was approved by the Universidad de los Andes and Pan American Health Organization ethics committee in their Acts No. 658 of 2016 and 2017-04-0042 of 2017, respectively.

Powered by Editorial Manager® and ProduXion Manager® from Aries Systems Corporation

**Format for specific study types**<br><br>**Human Subject Research (involving human participants and/or tissue)**<br>• Give the name of the institutional review board or ethics committee that approved the study<br>• Include the approval number and/or a statement indicating approval of this research<br>• Indicate the form of consent obtained (written/oral) or the reason that consent was not obtained (e.g. the data were analyzed anonymously)<br><br>**Animal Research (involving vertebrate animals, embryos or tissues)**<br>• Provide the name of the Institutional Animal Care and Use Committee (IACUC) or other relevant ethics board that reviewed the study protocol, and indicate whether they approved this research or granted a formal waiver of ethical approval<br>• Include an approval number if one was obtained<br>• If the study involved *non-human primates*, add *additional details* about animal welfare and steps taken to ameliorate suffering<br>• If anesthesia, euthanasia, or any kind of animal sacrifice is part of the study, include briefly which substances and/or methods were applied<br><br>**Field Research**<br><br>Include the following details if this study involves the collection of plant, animal, or other materials from a natural setting:<br>• Field permit number<br>• Name of the institution or relevant body that granted permission	
**Data Availability**<br><br>Authors are required to make all data underlying the findings described fully available, without restriction, and from the time of publication. PLOS allows rare exceptions to address legal and ethical concerns. See the PLOS Data Policy and FAQ for detailed information.	Yes - all data are fully available without restriction

A Data Availability Statement describing where the data can be found is required at submission. Your answers to this question constitute the Data Availability Statement and **will be published in the article**, if accepted.<br><br>**Important:** Stating 'data available on request from the author' is not sufficient. If your data are only available upon request, select 'No' for the first question and explain your exceptional situation in the text box.<br><br>Do the authors confirm that all data underlying the findings described in their manuscript are fully available without restriction?	
**Describe where the data may be found in full sentences. If you are copying our sample text, replace any instances of XXX with the appropriate details.**<br><br>• If the data are **held or will be held in a public repository**, include URLs, accession numbers or DOIs. If this information will only be available after acceptance, indicate this by ticking the box below. For example: *All XXX files are available from the XXX database (accession number(s) XXX, XXX.).*<br>• If the data are all contained **within the manuscript and/or Supporting Information files**, enter the following: *All relevant data are within the manuscript and its Supporting Information files.*<br>• If neither of these applies but you are able to provide **details of access elsewhere**, with or without limitations, please do so. For example:<br><br>*Data cannot be shared publicly because of [XXX]. Data are available from the XXX Institutional Data Access / Ethics Committee (contact via XXX) for researchers who meet the criteria for access to confidential data.*<br><br>*The data underlying the results presented in the study are available from (include the name of the third party*	All relevant data are within the manuscript and its Supporting Information files.

and contact information or URL).<br>• This text is appropriate if the data are owned by a third party and authors do not have permission to share the data.<br><br>* typeset	
Additional data availability information:	

Manuscript

Click here to access/download;Manuscript;Vivencias de mujeres con Virus del Zika Final Version Ing.docx

Experiences of women with ZIKV virus (ZIKV) versus the provision of health services in two cities in Colombia: a qualitative study

Jovana A. Ocampo Cañas [1¶*], Maria Yaneth Pinilla [1¶], Clemencia Navarro [1&], Carlos Mejia-Arbelaez[1&], Jhon Sebastián Patiño Rueda [1&]

[1] SIGIT Line-SEP Research Group, School of Medicine, Universidad de los Andes, Bogotá-Colombia

[*]Corresponding author

Email: ja.ocampo@uniandes.edu.co (JAOC)

[¶]These authors contributed equally to this work

[&]These authors also contributed equally to this work

Abstract

Background: In February 2016, the World Health Organization (WHO) declared the epidemic of the ZIKA virus (ZIKV) in Latin America to be a public health emergency. In Colombia, 11,944 pregnant women registered a ZIKV infection during the epidemic. So far, little is known about the experiences of women infected with ZIKV during their pregnancy, especially those relating to the provision of health services during the period of the epidemic.

Objective: To explore the experiences of pregnant women diagnosed with ZIKV infection about the provision of health services in two Colombian cities, considering the perspective of sexual and reproductive rights.

Methods: Qualitative study under the grounded theory approach, which uses semi-structured interviews as tools to explore the biographical experience of mothers during their gestation process and ZIKV infection, dividing the interview into two broad categories: before and during pregnancy.

Results: Twenty-two women were interviewed, 10 in the city of Cali and 12 in the city of Villavicencio. Information related to health service delivery during the ZIKV epidemic was obtained. In addition, we analyzed data on the transformation of their pregnancy experience when they were infected with ZIKV. Data were also obtained on the assimilation of the news received during pregnancy, the impact of the products of gestation on their lives and in the dynamic of their family, and, lastly, the social and cultural aspects, which determined the level of autonomy of women in the exercise of their sexual and reproductive rights, beyond the context of the epidemic.

Conclusions: In the health care of ZIKV epidemics, it is necessary to include the gender perspective, more specifically, sexual and reproductive rights. In addition, these epidemics must

be addressed through a comprehensive, appropriate, and not fragmented health system, in which sexual and reproductive rights must be mainstreamed in all health promotion and prevention programs.

Introduction

In February 2016, the World Health Organization (WHO), motivated by the increase in the incidence of microcephaly in newborns in Brazil [1–3], declared the ZIKA Virus (ZIKV) epidemic in Latin America as a Public Health emergency and, therefore, an emergency of international concern [4–6].

In Colombia, the behavior of the disease was similar to that of the countries that have areas or zones located below 2,200 meters above sea level (masl), reporting, between August 2015 and April 2016, 11,944 confirmed pregnant women with ZIKV, of which 12% (1,484 cases) were positive in laboratory tests (RT-PCR) [7,8].

The ZIKV was distributed in much of the Colombian territory, affecting cities such as Cali, Valle del Cauca (1,018 masl) and Villavicencio, Meta (467 masl) [7,9]. These two departments occupied, respectively, the first and eighth place in case reporting (suspected and confirmed) in the general population with ZIKV, and, ranked first and seventh in reporting of pregnant women [7,9]. In addition, they were characterized by having a good surveillance system and incorporating the care protocols issued by the World Health Organization (WHO / PAHO) [10], the Colombian Ministry of Health and the National Institute of Health [11].

In Cali, 1,259 cases were reported with an average age of 26 years. Of the total cases, 248 were confirmed by laboratory test (19.7%), 992 by clinical tests (78.79%) and 19 were classified as suspect (1.51%) [12]. Likewise, 27 cases of death before childbirth were reported in the products

of pregnancy. In Villavicencio, 449 cases were reported, of which 245 (54.6%) were confirmed by lab tests, 204 (54.4%) confirmed by clinical tests, and there were no suspected cases [12].

In these two cities, as in the rest of the country and other Latin American cities, efforts were made to implement all the promotion and prevention actions in the pregnant population, with the aim of reducing the presentation of cases of microcephaly. These actions were stipulated by the WHO and the Colombian Ministry of Health [10,11]. However, it is possible that such actions have not been sufficient due to multiple elements, including the lack of integration by health areas, such as the area of Vector Borne Diseases (VTDs) and the area of Sexual and Reproductive Health, or the lack of a comprehensive care approach geared to the needs of patients that includes a gender approach, guaranteeing the exercise of their sexual and reproductive rights, both from public policy point of view and from the provision of health services [13–15].

So far, little is known about the experiences of women infected with ZIKV during pregnancy, especially in relation to the provision of health services during the period of the epidemic, so there is a knowledge gap that needs to be explored. This study is aimed to explore the experiences of pregnant women diagnosed with ZIKV infection, with regard to the provision of health services in two Colombian cities, to understand, in this way, how their transit was in the Colombian health system, taking into account the framework of sexual and reproductive rights.

Materials and methods

Study design

With the aim of making an approach to the experiences of pregnant women infected with ZIKV regarding the provision of health services, a qualitative study was conducted under the approach of the Grounded Theory [16,17]. Using semi-structured interviews, which were analyzed through

a comprehensive and dynamic coding process that prioritized the discovery of emerging codes, we identified relevant elements to understand the problem from a social and cultural perspective, through the constant analysis and comparison of these discourses, as well as the construction of analytical categories.

In each one of these moments (before and during pregnancy), it was possible to determine the provision of specific health services received by the pregnant woman. This allows the analysis of these experiences not only to be related to the specific time of the ZIKV involvement, but, in addition, incorporates elements that allow us to understand how they interacted with the health system according to their social context and living conditions.

Ethical considerations

The investigation was carried out under the criteria of resolution 008430 of October 4, 1993 of the Republic of Colombia [18], according to which it is characterized as "without risk" by being immersed in the category of studies using retrospective documentary research techniques and methods and, where no intentional intervention or modification is performed of the biological, physiological, or psychological or social variables of the individuals. Similarly, respect for human dignity was maintained and informed consent was obtained, in which aspects such as the objective, methodology, handling of the information provided, results, disclosure of the findings and the right to reply or not to questions and suspend the interviews when the participants so wish was observed. The protocol of the study was approved by the Universidad de los Andes and Pan American Health Organization ethics committee in their Acts No. 658 of 2016 and 2017-04-0042 of 2017, respectively.

Sampling and selection of participants

The information on the reported cases of ZIKV was provided by the Municipal Health Secretariats of Cali and Villavicencio, through the SIVIGILA (National System of Public Health Surveillance) information system.

Two databases were obtained; one from pregnant women registered as suspected or confirmed cases of infection with ZIKV and another from congenital defects that entered the health system between 2015 and 2017. Both databases were reviewed to identify women who had been reported as ZIKV cases, who had a possible outcome of a newborn with microcephaly, and who lived in the cities of Cali and Villavicencio. We identified 39 women who were pregnant, registered as suspected or confirmed cases, and was recorded as a possible outcome with microcephaly.

The invitation to participate in the study was made by telephone, with the support of the Municipal Health Secretariats. Of the 39 women contacted, 22 agreed to participate, 5 refused and 12 could not be contacted due to incorrect registration data (Fig 1)

Fig 1. Enrollment Flow Diagram

Interviews were carried out in 2018, in the period after pregnancy. Four professional interviewers in anthropology, sociology, medicine, and nursing were involved during the field work phase of the study and received prior training in the methodological characteristics of the interview. These trainings included knowledge of the instrument, ethical aspects, and informed consent, as well as instructions for recording and handling information.

Interview guide

The instrument was organized according to the experience gained by the women in two moments: the first period was labeled "**before**", in which enquiries are made about the structure and family

environment of the interviewee, as well as the situation of the context of pregnancy (e.g.: planned or unplanned pregnancy) and on sexual and reproductive health habits prior to the pregnancy

The second period, **during** pregnancy, in which two central elements were investigated: the experience of pregnancy and the health care received. This last element focused on aspects such as the quality of care received (understood from availability, acceptability, accessibility, and professional suitability [19]), screening test, treatments and recommendations given. Table 1

Table 1 Structure of the Interview Guide

Guide segments	Description
Characterization data	General information and basic data of the interviewee for characterization.
Family context at the time of pregnancy	Inquiries about the structure and family environment of the interviewee as well as the context of her pregnancy. It also ascertains whether the pregnancy was planned or not, as well as the sexual and reproductive health habits prior to the pregnancy.
Pregnancy	Investigate for pregnancy detection, reactions of the woman and the family.
Health care	Inquiries about the time when subject goes to the health service, causes and first interaction with health personnel during pregnancy.
Paraclinical studies, ultrasound, and medications	Inquiries about specific tests (ultrasound, labs, cytology) and times.
Times in care	Inquiries about control appointments to review results and specific moments of health care attention.
Zika	Investigates all aspects related to Zika, from the first-time subject hears about the epidemic, knowledge, imaginaries, access to information or prevention campaigns, diagnosis, reaction of partner and family, personal process, and related experiences.

Continuation of pregnancy	Depending on the outcome, reasons why subject decides to continue with pregnancy, childbirth, and post-natal situation. If born alive, inquiries about the child as well as changes in personal and family dynamics.
Miscarriage - Elective abortion	Depending on the outcome, reasons why subject decided for the elective abortion, support received, experiences of the process and subsequent perception.
Conditions of home	Aspects related to the environment where the house is located (possible sources that generate risk of contact with the vector) and conditions of access to public services, health services and general conditions.
Information received	Information received during the epidemic such as training, participation in contingency plans, advisories.
Care process	Description of the health care process, inquiry into perception regarding the service, difficulties, positive aspects, recommendations, approach by health personnel of topics such as diagnosis and elective abortion.

The pilot application of the interview guide was conducted in the first quarter of 2018 to four women, who had been pregnant in the period of the epidemic and were suspected of ZIKV infection. However, in the bases initially delivered, these reported different outcomes than microcephaly, among which were: healthy child, voluntary termination of pregnancy (VTP), perinatal mortality, among others. No modification or adjustment was necessary after the application of the pilot test.

Data collection

The interviews were conducted in the second and third quarters of 2018. The meeting places for the development of these were arranged according to the availability and preference of the interviewees: at home, at their workplace or in a nearby public place. In the interviews conducted

at home, several participants were accompanied by their partner or parents, who were asked to not interact during the recording.

Once the informed consent was accepted, the audio recording of the interviews was made for subsequent transcription. The duration of the interviews varied according to aspects such as: the availability of time for women and their willingness to speak about some topics (death, VTP, later illness of the child). In addition, these interviews were supplemented with notes in field journals on other aspects of context, as well as comments that were not captured in the recordings. The interviews were transcribed in their entirety and a process of anonymization and review of the transcripts was carried out to ensure security, fidelity, and quality of the content.

Subsequently, a multidisciplinary team was formed for the coding and analysis phase, which included four researchers and four final-year medical students. A first review was made, selecting a group of interviews, with the aim of integrating the issues raised in the initial guide with an identification of emerging issues. Also, a code structure was created to be used by the encoders. The coding and analysis process were carried out with the support of the QSR NVivo 12® software, facilitating the review and merger of projects, as well as the export of reports for the analysis of results (S1_Fig).

Limitations of this study

Because the data selected for this study were reported to the SIVIGILA, some of the case experiences, which were not reported through this system, could be left out of this study.

Additionally, as already mentioned, out of the 39 identified cases, we were unable to contact 12 of them due to lack of information, or erroneous information in the registry. The aforementioned

generates the risk of leaving out experiences of women other than those exposed in this study and, therefore, a variation in the results obtained.

Findings and discussion

It is important to emphasize that the discussion presented is a retrospective exercise of the women who participated in this research, who have elaborated and reworked their stories based on their experience at that time, a process that they continue to carry out, since it marked the course of their lives today, leaving them, with the passage of time, anchored to the ZIKV.

Characteristics of the participants

In total, 22 women participated, 10 in the city of Cali and 12 in the city of Villavicencio. S1 and S2 Tables summarize the characteristics of the women interviewed in each city and present the identification of each interview, which will be used to reference the textual citations presented in the results.

The average age of pregnancy was 27.6 years. Regarding the level of education, all the women interviewed reported having basic primary education, 10 of them completed basic secondary education and 11 reported having a higher education; only one of them reported incomplete basic secondary education.

Regarding the socio-economic level, 3 women interviewed were in a low socio-economic level, 15 were in a medium socio-economic level and 4 women in a high socio-economic level. About the health system membership scheme, 16 had an employer-based insurance scheme (contributory scheme), 5 had a subsidized scheme paid for by the health system (subsidized scheme), and 1 belonged to the special scheme.

Half of the interviewees had some type of paid employment relationship, either as wage earners or self-employed. Six of them were engaged in household work or occasional work, for which they did not receive, or sporadically received, some form of remuneration. Only one woman stated that she was unemployed and three of them were university students at the time of pregnancy.

When returning to the aspects addressed in the interview guide, the categories of analysis were analyzed during the two moments previously mentioned (before and during pregnancy.)

Before pregnancy

Contraception and promotion-prevention activities

Here are the promotional and preventive activities received by women regarding contraception before pregnancy.

Of the 22 women interviewed, 17 were not planning when they became pregnant. Some of them said they were not planning regularly for various reasons, including: considering that they did not have an active or very occasional sexual life, experiencing discomfort with some methods such as pills and injections; which led them to be inconsistent in the use of these methods. These reasons are related to the reasons reported in the literature on the non-use of contraception or discontinuation thereof [20–24].

> *(...) We have always planned the same, nothing else. First, because we don't like it, because the injection and all contraceptives hurt me (...), we have always planned in a natural way and so, simply put, but they do tell you "plan with such a thing": But really, we´ve never listened.* [**Interviewee 7]**

The 22 women were already pregnant at the time of hearing prevention warnings about the epidemic. Seventeen of them did not know it because they were unplanned pregnancies.

When asked about how to learn about ZIKV prevention strategies, the media, mainly television, were the most reported by women. In this case, they mentioned having heard, in addition to the risks for pregnant women, the recommendations for the use of repellent and mosquito netting to prevent the epidemic.

> *(...) (...) Before I became pregnant, I had already felt that the ZIKV was what they said in the news. Then, that the mosquito ... and then I consulted, I mean, days before I knew that I was pregnant. I went to the Employer-based health insurance (EHS) because I felt like awful, and I had welts on my body, I went to the doctor. I also had a fever, so I told him that maybe it was that the ZIKV, so I consulted. But I did not know I was pregnant, when I took the exams to find out if it was ZIKV. And so, between those exams, I found out I was pregnant and it came out positive, I mean, I took the exams in the hospital (...) I do not remember (...)* [**Interviewee 1]**

An analysis of what women say in this area shows that, for various reasons, they were not planning regularly in the time before pregnancy or had poor advice on contraception. Data contrasted with the 2015 National Demography and Health Survey, which shows that 82.5% of sexually active women use some form of contraception [24].

Economic constraints were also mentioned, with many women paying for their own planning methods, making the lack of economic resources a barrier to the use of contraceptive methods [25–27]. Therefore, a public policy that allows free access to contraceptive methods would facilitate the reduction of this economic barrier, which would increase the effective rate of contraception [28,29].

Mention was also made of social and cultural constraints, such as the lack of involvement of men in sexual and reproductive health issues and the fact of exclusively holding women responsible for issues related to contraception, which contrasts with their lack of autonomy to decide whether or not to have protected sex with her partner [30,31].

In the light of the above, it was found in the experiences of women that there was a lack of effective strategies to contribute to their sexual and reproductive health before the epidemic.

From the health care received, the information on pregnancy prevention was addressed, almost exclusively, to women. On many occasions, this information was predominantly targeted at women travelling to endemic areas and not at the inhabitants of these territories [32].

In other cases, ZIKV was associated with diseases that had already had an incidence in the area, such as Dengue and Chikungunya, which made it unaware of its severity, as it was not considered fatal or high-risk. These data are consistent with the results of other studies, where the relationship between these three tropical diseases and the community's perceptions about their symptoms and severity was evidenced [33,34], causing the ZIKV and its possible perinatal outcomes to be undervalued [33].

In turn, several of the information campaigns that were carried out had a more reactive than preventive component and, therefore, many spaces designed to inform about care and prevention during pregnancy were generated on a temporary basis, in response to proliferation of ZIKV infection. As a result, it was evident that these campaigns were not conceived prior to the epidemic, or as part of long-term strategies and programs.

On the other hand, the prevention campaigns were aimed at vector control through fumigation, the use of repellent and mosquito nets, and only a part was destined to recommendations to postpone

pregnancy and to use protection measures [32]. This reaffirms that these campaigns were carried out, in essence, from the perspective of risk factors: risk practices or behaviors.

However, one of the problems with such approaches is that the efforts "are hampered, as the proposals can be translated into health programs that lack coherence in the particular contexts of the communities or a practical vision that allows for a viable application within the community" [35].

During pregnancy

The number of women whose pregnancies were unplanned coincided with the number of women who were not planning (17 women). Being unplanned pregnancies, the news of this already had an implication for women and their families.

> ***P:*** *No, because it gave me a lot of happiness and at the same time, I was afraid, really, because it is a total life change, this is what I felt.* ***E:*** *Afraid just because of the change or for something else?* ***P:*** *No, because of the change of life, because one hopes, but then, at that time, I did not imagine it.* ***E:*** *Who did you tell?* ***P:*** *My husband.* ***E:*** *What did your husband tell you?* ***P:*** *No nothing because this news really gave him a lot of joy. I woke him up that day telling him that news, he gave him a lot of joy*. [**Interviewee 9]**

Regarding the symptoms of ZIKV, 2 women reported no symptoms, 12 had symptoms in the first trimester of pregnancy, 5 in the second trimester and 3 in the third trimester. The average number of days between the onset of symptoms and your initial consultation was between 3 to 5 days, with their initial consultation being attended, in 16 of the cases, by a general doctor in the emergency service, the other 6 consultations having been attended by outpatient services or specialized consultation.

> *(...) I was in the autoclaves, I got into the autoclave and I left and after about two hours I started to break out all over, a rash on my face and body, and I told him, suddenly it might have been that I touched something the autoclave and that gave me the allergy. But I did not run a fever, nor did my eyes get red; it did not give me anything, none of that, but I did start the pregnancy with a migraine. So, the only thing they prescribed me was acetaminophen; and that night I went to the ER, because the outbreak spread to my stomach and everything. I went there and what the boss did was scold me because I was pregnant at a time when the ZIKV epidemic was around. I told him that it was not by work and grace, but it was as it is said, it was not planned; So well, they sent me the ZIKV tests and, from there, they sent me to the laboratory and I told them that I only had taken acetaminophen.* [**Interviewee 17]**

Regarding the biographical experience of pregnancy when affected by ZIKV, emphasizing the process of quality of the health care (diagnosis-treatment and promotion and prevention actions) the following is evident:

> *(...) And supposedly the doctor who was seeing her, the baby girl, was growing normal, because they even told me to sue that clinic because I paid all the private ultrasounds to him, and according to him the baby girl was growing normal, until the eighth month. That she had a problem and I had to go to the ER and it was there when the guy who did the ultrasound there, said "no, the girl comes with microcephaly". That same day, we went to the doctor whom I was paying, and he did the ultrasound and he also had a transvaginal and said "no, the girl ...", I said "doctor, but how can this be possible, I am keeping track of (this pregnancy with) you, I have already had four ultrasounds with you, monthly, and*

you tell me that it is normal child. And how is microcephaly going to occur in less than 20 days? [**Interviewee 10]**

About the provision of health services, in terms of the quality of medical equipment or supplies, the women indicated that these were not sufficient, adequate, or appropriate. Added to this is the suitability of the medical professional, in terms of knowledge and management of the ZIKV epidemic, since they did not have a deep understanding of the guide and limited specific actions here and now, without achieving the articulation of the interventions or with their peers.

…Well, the bad truth, because well, one will always ask for a control and there never is (one available), unless suddenly, as one asks, asks now as a very extreme favor, because there are almost never any appointments available… and really, well, I was worried at that time that I had that Zika but then I never received (got) a ..., Now I think back and say I never received a visit, or a call or something, a follow-up that would tell me…[**Interviewee 12]**

On the subject of the information received, both in the diagnosis and in later moments, one of the aspects that could have influenced this was the lack of better coordination between the different actors in the health sector, regarding protocols, procedures and communication channels of the epidemic, which caused each one to fulfill the assigned tasks without understanding the complexity of the phenomenon [36].

…A head of the Meta clinic called me and she said "oh Mrs. Merly, I have to inform you that result of the Zika report came positive", because I did get angry, because I was already so advanced with my pregnancy. She tells me that I had Zika, and I started to get scared about the head, that microcephaly, everything they tell you. So when they gave me that report, anyway, the gynecologist gave me a monthly ultrasound, so ... after that, my blood

> *pressure started to rise and I had an ultrasound every month to see that the baby would not come. So, when they told me about the Zika, then at once they gave me an appointment with the gynecologist. I gave him the report, at once he sent me to check-ups, whenever he did the ultrasound scans of the baby, the brain girth and all the baby's arteries were fine. So hence the doctor began to do them month by month, and…* [**Interviewee 17]**

This is reflected in the often-confusing information received by woman and the late conduct and interpretation of tests and examinations. These delays had significant and negative consequences for the women, as well as the sense of fear, uncertainty and guilt based on the information received about their state of health.

Similarly, it was evident that the care provided, both in public and private health services, was characterized by being distant from the emotions and needs of pregnant women. The pain of this dehumanized treatment is a constant mark in each of the women's stories.

> *…Then a doctor came and offended me. He told me "because you can go home divinely, because you are not sick, you are going to have your normal child, your child, although it has a complication, is something that can be normal" So he said "what do you say?" stay eating and sleeping here. If that's what you want, then stay ". I said," why are you talking to me like that?" I said, "I'm not going to make decisions for me, because I did not study for that…* [**Interviewee 20]**

In addition, the channels of communication in the epidemic were not clear, the responsibilities of each of the actors (State, insurers and providers), were not specific enough and, in the territory, the articulating axis was overwhelmed in terms of these [36]. Of course, each of the actors did everything possible to fulfill the assigned task without understanding the complexity of the phenomenon, in response to the protocols and implemented guides.

The counseling process of the Voluntary Termination of Pregnancy (VTP), offered by the health personnel, generated in the women a lot of fear because, in addition, they only offered them the procedure, but they did not explain why or what for, and what other alternatives were there in women. This made many of them, upon hearing the information from the health personnel, leave with more doubts, which delayed the decision-making.

> *…No, never, never, the same as in the time that I already had, I was already more than 16 weeks old, so no, no, the same, she never… no. What she told me was, I think she wanted, that should calm down because I was very distressed that day, I went into a crisis that day and I held on to crying. I was very distressed because I knew what was happening. So, I think what she did was try to reassure me, and they sent me to the other ultrasound and from there to there, every month they did an ultrasound.…* [**Interviewee 8]**

Furthermore, decisions were influenced by a high religious content and denial of autonomy, of women, for decision-making. In none of the cases was the mother asked if she had wanted her child, if it was a planned pregnancy and if she wanted to continue with or terminate the pregnancy. It was the doctor who made the decision for the woman or, even, committees were created in some hospitals to decide whether to approve the VTP.

> *No, because there she had to, with that ultrasound, it was already defined if, since she already was 7 and a half months pregnant, right? Then, from there, it was already defined what the gynecologist said in that ultrasound, he defined whether to terminate the pregnancy, I had to go to a meeting, to a medical meeting…* [**Interviewee 3]**

Regarding sexual and reproductive rights, although women do not explicitly mention them, in their interviews it is evident that they are not applied in different scenarios:

> *(...) And so, well, when I left, there they could process me and that. There they said they were not going to interrupt my pregnancy, that I should have come with some pain, yes, "do you not understand that I came for a termination of pregnancy?”. I said that I came with a child with hydrocephaly, and I told him that I did not want the baby, I told him that I did not want that child, that it needed to be removed as quickly as possible; so, they hospitalized me. What day did they hospitalize me? I don't know, I know I stayed in the hospital for 6 days.* [**Interviewee 18]**

> *(...) Yeah, also that this was kind of bad. And already, that day, the same perinatologist was called, the doctor, told me that this pregnancy ..., he did tell me, not the doctor but the one who did the ultrasound told me that this pregnancy was of high risk, that it could last 4 months and it was coming, that it was better for him to make the decision and me not have the baby. But I told him no, that I wanted to have my baby because there are people who tell him that, and I had a little niece who was told that, and my niece is normal, so that's why, and she told me it was better not to have it.* [**Interviewee 6]**

Regarding recommendations on the health care process

Women’s main recommendations include an increased awareness of ZIKV infections as an epidemic among both institutions and the community in general.

In addition, the need for greater priority attention is evident, where the information is provided in a clear and massive way, beyond advertising, because in the environment there is a lot of ignorance about how to treat and cope with the disease.

Also, some women suggest prompt care, since any delay affected the lives of mothers and their unborn children. Finally, women highlight the improvement of their treatment of them during any contact with health services.

> *(...) To say the least, I would say that they should be more competent in that sense, and not make these mothers feel bad; treat them with more love. I think that, if they are doctors, they have a lot of ethics, and they should be people more vulnerable (sic) with those mommies, those girls who ..., because there are many girls who are very young, there are others who are so far with their first child and are more like that. Doctors should be less rude, less lout with these girls, and I have always said that if the mother chooses, they are not the ones who are going to carry that obligation, yes? Then, don't make them feel bad and don´t make that person feel as if she were a strange bug, because it is a life we are talking about, a life, come as it comes, and let's go on, yes? And so, we are talking about a life, we are talking about a person who also had a life and not because we were ... well...*, *then everyone has to be ... well ... like us. This is what life is; I would ask for more than respect, I would ask more respect and more vulnerability of them, so that they are not so run over, that the things that they are going to do to them are more expedited, much faster, the attention to these pregnant mothers on this issue.* [**Interviewee 1]**

Conclusions

As the objective of the research is to explore the experiences of pregnant women, diagnosed with ZIKV infection, with regard to the provision of health services, one of the central elements for this approach is their biographical experience, which covered not only the aspects related to the provision of the service as such, but also the transformation of the experience of their pregnancy when affected by ZIKV infection, the assimilation of the news received during pregnancy, the impact on their lives and that of their families as a result of the different outcomes and, finally, the social and cultural aspects that determine the women's level of autonomy in the exercise of their sexual and reproductive rights, beyond the context of the epidemic.

When analyzing the information collected, it can be concluded that many factors determined the experiences of pregnant women in the ZIKV epidemic, which in one way or another changed their lives forever. These factors began from the moments before conception and continued to interfere throughout the pregnancy, making this experience something they would not want to repeat again.

Since this investigation revealed that there was a violation of the sexual and reproductive rights of the women interviewed, who were not able to make autonomous decisions about their bodies (contraception, voluntary termination of pregnancy), loss or abandonment by their life partners at the time of birth of their children, as well as in the changes in family dynamics and job abandonment, which led them to give up immediate dreams.

In addition, there was evidence of loneliness and abandonment on part of the health sector, obstetric violence, non-inclusion by men for the joint care of their partners throughout the pregnancy process, poor psychosocial care, fear of stigma for having a child with some type of

congenital malformation, punishment for deciding to terminate their pregnancy and moral judgment, by family members and health personnel, for carrying out the elective abortion.

Additionally, according to the women's view, the minimum standards of care were not guaranteed under the current health system. Although care protocols were complied with, these were not in the times required by the women, in the context of their illness, since examinations and images were not appropriate in the context of the epidemic, creating harm for them and their unborn children.

The foregoing also shows that, despite the existence of a sexual and reproductive health policy, it has not yet succeeded in Colombia in incorporating the needs of women.

In general terms, and in accordance with the objectives of the study, the contributions or fundamental findings obtained in the study are related to the need to strengthen the gender perspective of the ZIKV epidemic, approaching this epidemic from a health system that is not fragmented, comprehensive and appropriate, and that sexual and reproductive rights must be mainstreamed into all promotion and prevention programs.

Acknowledgments and funding

We want to thank the women and their families of Cali and Villavicencio, who opened their hearts to us to share their experiences. We also want to thank the work teams of Public Health, Epidemiological Surveillance of the Health Secretariats of Villavicencio, especially Alexandra Pardo and, in Cali, to Javier Colorado. In addition, we appreciate the support of the epidemiological surveillance team, ETV group, of the National Institute of Health of Colombia, and to the members of the SIGIT research line of the SEP group of the Faculty of Medicine, Juliana Zambrano, María Canal Caicedo, Angélica Carolina Gutiérrez Cifuentes, Andrés Mauricio García, Álvaro Ayala, and Andrés Fidel Moreno.

References

1. Zorrilla CD, García García I, García Fragoso L, De La Vega A. Zika Virus Infection in Pregnancy: Maternal, Fetal, and Neonatal Considerations. J Infect Dis. 2017. doi:10.1093/infdis/jix448

2. Lin HZ, Tambyah PA, Yong EL, Biswas A, Chan SY. A review of Zika virus infections in pregnancy and implications for antenatal care in Singapore. Singapore Medical Journal. 2017. doi:10.11622/smedj.2017026

3. Cauchemez S, Besnard M, Bompard P, Dub T, Guillemette-Artur P, Eyrolle-Guignot D, et al. Association between Zika virus and microcephaly in French Polynesia, 2013-15: A retrospective study. Lancet. 2016. doi:10.1016/S0140-6736(16)00651-6

4. World Health Organization (WHO). WHO statement on the first meeting of the International Health Regulations (2005) (IHR 2005) Emergency Committee on Zika virus and observed increase in neurological disorders and neonatal malformations. [cited 3 Dec 2020]. Available: https://www.who.int/news/item/01-02-2016-who-statement-on-the-first-meeting-of-the-international-health-regulations-(2005)-(ihr-2005)-emergency-committee-on-zika-virus-and-observed-increase-in-neurological-disorders-and-neonatal-malformations

5. Teixeira MG, Da Conceição N Costa M, De Oliveira WK, Nunes ML, Rodrigues LC. The epidemic of Zika virus-related microcephaly in Brazil: Detection, control, etiology, and future scenarios. Am J Public Health. 2016. doi:10.2105/AJPH.2016.303113

6. Redondo Bravo L, Suarez Rodríguez B, Fernández Martínez B, Simón Soria F, Díaz García O, Sierra Moros MJ. [Zika virus epidemic. The Public Health response in Spain]. Rev Esp Salud Publica. 2018.

7. Instituto Nacional de Salud. Boletin Epidemiológico Número 52 de 2016. Boletín Epidemiológico Sem. 2016;52: 50–52.

8. Pacheco O, Beltrán M, Nelson CA, Valencia D, Tolosa N, Farr SL, et al. Zika Virus Disease in Colombia — Preliminary Report. N Engl J Med. 2020. doi:10.1056/nejmoa1604037

9. Instituto Nacional de Salud -INS. Boletin Epidemiológico Semanal. Semana epidemiológica │número 29 de 2016│ 17 julio - 23 julio. 2016;27: 1–110. Available: https://www.ins.gov.co/buscador-eventos/BoletinEpidemiologico/2016 Boletín epidemiológico semana 29.pdf

10. Pan American Health Organization. Guideline for surveillance of Zika virus disease and its complications. Washington D.C.; 2016. Available: https://iris.paho.org/bitstream/handle/10665.2/28405/9789275118948_eng.pdf?sequence=1&isAllowed=y

11. Ministerio de Salud y de Proteccion Social. Lineamientos Provisionales Para El Abordaje Clinico De Gestantes Expuestas Al Virus Zika En Colombia. Febrero. 2016; 35. Available: https://www.minsalud.gov.co/sites/rid/Lists/BibliotecaDigital/RIDE/VS/PP/ET/lineamientos-provionales-abordaje-clinico-gestantes-expuestas-zika-colombia.pdf

12. Ocampo Cañas JA, Caviedes Combita D, Molina Leon HF, Garcia Sierra AM, Hernández Florez LJ. Patient characteristics and pregnancy outcomes among Zika-infected pregnant women: Epidemiologic surveillance data from two cities in Colombia, 2015–2016. Int J Gynecol Obstet. 2020. doi:10.1002/ijgo.13041

13. Filgueiras Meireles JF, Neves CM, Morgado FF da R, Caputo Ferreira ME. Zika virus and pregnant women: A psychological approach. Psychol Heal. 2017. doi:10.1080/08870446.2017.1307369

14. Ebuenyi ID, Bhuyan SS, Bain LE. Zika virus infection and microcephaly: Anxiety burden for women. Pan African Medical Journal. 2018. doi:10.11604/pamj.2018.30.2.11794

15. Arenas-Monreal L, Piña-Pozas M, Gómez-Dantés H. Challenges and inputs of the gender perspective to the study of vector borne diseases. Salud Publica Mex. 2015. doi:10.21149/spm.v57i1.7404

16. Gaete Quezada RA. Reflexiones sobre las bases y procedimientos de la Teoría Fundamentada. Ciencia, Docencia y Tecnol. 2014.

17. Glaser BG, Strauss AL. Discovery of grounded theory: Strategies for qualitative research. Discovery of Grounded Theory: Strategies for Qualitative Research. 2017. doi:10.4324/9780203793206

18. Ministerio de Salud y de Protección Social. Resolución Número 8430 de 1993. Ministerio de Salud y Protección Social 1993.

19. Kerguelen C. Calidad en Salud en Colombia. Universidad Nacional de Colombia. 2008. Available: https://www.minsalud.gov.co/Documentos y Publicaciones/CALIDAD EN SALUD EN COLOMBIA.pdf

20. Bellizzi S, Mannava P, Nagai M, Sobel HL. Reasons for discontinuation of contraception among women with a current unintended pregnancy in 36 low and middle-income countries. Contraception. 2020. doi:10.1016/j.contraception.2019.09.006

21. Bellizzi S, Palestra F, Pichierri G. Adolescent Women with Unintended Pregnancy in Low- and Middle-Income Countries: Reasons for Discontinuation of Contraception. J Pediatr Adolesc Gynecol. 2020. doi:10.1016/j.jpag.2019.11.004

22. Simmons RG, Sanders JN, Geist C, Gawron L, Myers K, Turok DK. Predictors of contraceptive switching and discontinuation within the first 6 months of use among Highly Effective Reversible Contraceptive Initiative Salt Lake study participants. Am J Obstet Gynecol. 2019. doi:10.1016/j.ajog.2018.12.022

23. Weldemariam KT, Gezae KE, Abebe HT. Reasons and multilevel factors associated with unscheduled contraceptive use discontinuation in Ethiopia: Evidence from Ethiopian demographic and health survey 2016. BMC Public Health. 2019. doi:10.1186/s12889-019-8088-z

24. Ministerio de salud y proteccion social; profamilia. Encuesta Nacional de Demografía y Salud Componente de Salud Sexual y Salud Reproductiva 2015. 2015.

25. Cartwright AF, Otai J, Maytan-Joneydi A, McGuire C, Sullivan E, Olumide A, et al. Access to family planning for youth: Perspectives of young family planning leaders from 40 countries. Gates Open Res. 2019. doi:10.12688/gatesopenres.13045.2

26. Wolgemuth TE, Cuddeback M, Callegari LS, Rodriguez KL, Zhao X, Borrero S. Perceived Barriers and Facilitators to Contraceptive Use Among Women Veterans Accessing the Veterans Affairs Healthcare System. Women's Heal Issues. 2020. doi:10.1016/j.whi.2019.08.005

27. Eisenberg D, McNicholas C, Peipert JF. Cost as a barrier to long-acting reversible contraceptive (LARC) use in adolescents. Journal of Adolescent Health. 2013. doi:10.1016/j.jadohealth.2013.01.012

28. Di Meglio G, Yorke E. Universal access to no-cost contraception for youth in Canada. Paediatr Child Heal. 2019. doi:10.1093/pch/pxz033

29. Snyder AH, Weisman CS, Liu G, Leslie D, Chuang CH. The Impact of the Affordable Care Act on Contraceptive Use and Costs among Privately Insured Women. Women's Heal Issues. 2018. doi:10.1016/j.whi.2018.01.005

30. Bustamante-Forest R, Giarratano G. Changing men's involvement in reproductive health and family planning. Nursing Clinics of North America. 2004. doi:10.1016/j.cnur.2004.02.001

31. Sternberg P, Hubley J. Evaluating men's involvement as a strategy in sexual and reproductive

health promotion. Health Promotion International. 2004. doi:10.1093/heapro/dah312

32. Ministerio de Salud y Protección Social. Infografías 2016 Zika. [cited 3 Dec 2020]. Available: https://www.minsalud.gov.co/imagenes admcontenido/forms/thumbnails.aspx?rootfolder=/imagenes+admcontenido/infografias&folderctid =0x0120006336482bdf9f1c43ac06cd43c156fa84&view=%7Bbb8250cf-676b-445a-b101-8e8be7542e97%7D

33. Tirado V, Morales Mesa SA, Kinsman J, Ekström AM, Restrepo Jaramillo BN. Women's reluctance for pregnancy: Experiences and perceptions of Zika virus in Medellin, Colombia. Int J Gynecol Obstet. 2020. doi:10.1002/ijgo.13046

34. Weldon CT, Riley-Powell AR, Aguerre IM, Celis Nacimento RA, Morrison AC, Oberhelman RA, et al. 'Zika is everywhere': A qualitative exploration of knowledge, attitudes and practices towards Zika virus among women of reproductive age in Iquitos, Peru. PLoS Negl Trop Dis. 2018. doi:10.1371/journal.pntd.0006708

35. Suárez R, Beltrán EM, Sánchez T. El sentido del riesgo desde la antropología médica: consonancias y disonancias con la salud pública en dos enfermedades transmisibles. Antípoda Rev Antropol y Arqueol. 2006. doi:10.7440/antipoda3.2006.05

36. Gomez HM, Mejia Arbelaez C, Ocampo Cañas JA. A qualitative study of the experiences of pregnant women in accessing healthcare services during the Zika virus epidemic in Villavicencio, Colombia, 2015–2016. Int J Gynecol Obstet. 2020. doi:10.1002/ijgo.13045

Supporting information

S1 Fig. Initial analysis categories

S1 Table. Demographic characteristics and identification of each one of the women interviewed in the city of Cali., Colombia.

S2 Table. Demographic characteristics and identification of each of the women interviewed in the city of Villavicencio, Colombia

S1 Appendix. Research Protocol

 Click here to access/download;Figure;Fig1.tif

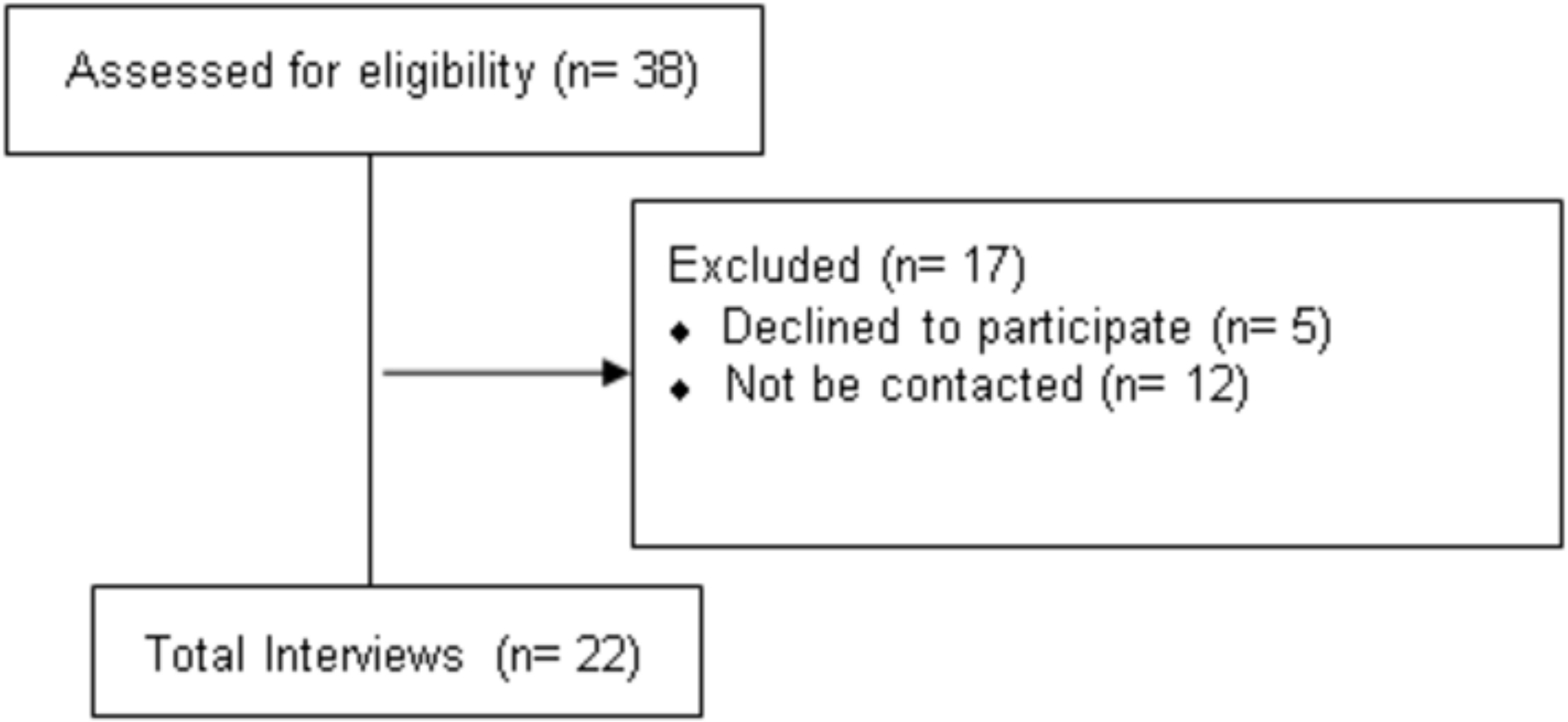

Appendix 1

Click here to access/download

Supporting Information

S1_Appendix.pdf

S1_ Fig

Click here to access/download

Supporting Information

S1_Fig.png

S1_Table

Click here to access/download
Supporting Information
S1_Table.pdf

S1_ Table

Click here to access/download
Supporting Information
S2_Table.pdf
