# Supplementary material for: Experiences of women with Zika virus (ZIKV) versus the provision of health services in two cities in Colombia: A qualitative study
Source: PLoS One. 2021 Dec 2;16(12):e0260583. doi: 10.1371/journal.pone.0260583 (PMC8638867; doi:10.1371/journal.pone.0260583)
Supplement: Attachment — Submitted filename: Rev.docx [file pone.0260583.s005.docx]

The manuscript by Campos et al addresses a highly pertinent topic and their findings/conclusions can be of significance in the field, the main aim was to explore the experiences of pregnant women, diagnosed with ZIKV infection, with regard to the provision of health services

The manuscript is very well written and structured. However, I have some minor observations

**Introduction**

The first two paragraphs are very short and with unfinished ideas (Unify)

Line 72 In Cali, 1,259 cases were reported with an average age of 26 years (A reference that supports this information is missing)

Line 74-75 Likewise, 27 cases of death before childbirth were reported in the products

of pregnancy (A reference that supports this information is missing)

In the paper Ocampo et al 2020 you have this information “Between April 2015 and August 2016, 11 944 pregnant women were reported to have Zika virus in Colombia, of which 12.4% (n=1484) were diagnosed positive using reverse transcriptase polymerase chain reaction (RT-PCR).”. That is very similar to the lines 61-64 in this manuscript. Please rephrase it.

Between April 2015 and August 2016, 11 944 pregnant women were

reported to have Zika virus in Colombia, of which 12.4% (n=1484)

were diagnosed posive using reverse transcriptase polymerase chain

reacon (RT- PCR

Between April 2015 and August 2016, 11 944 pregnant women were

reported to have Zika virus in Colombia, of which 12.4% (n=1484)

were diagnosed posive using reverse transcriptase polymerase chain

reacon (RT- PCR

**Materials and methods**

Figure 1. Have a mistake, assessed to elegibility should be 39 not 38 because 38 minus 17 is equal to 21 not 22

In introduction section you said (In Colombia, 11,944 34 pregnant women registered a ZIKV infection during the epidemic) Do you consider that 22 people are a significant sample? although it is mentioned in limitations.
